# Systems biology approach for mapping the response of human urothelial cells to infection by *Enterococcus faecalis*

**DOI:** 10.1186/1471-2105-8-S7-S2

**Published:** 2007-11-01

**Authors:** Mikhail G Dozmorov, Kimberly D Kyker, Ricardo Saban, Nathan Shankar, Arto S Baghdayan, Michael B Centola, Robert E Hurst

**Affiliations:** 1 Department of Urology, College of Medicine, Oklahoma University Health Sciences Center, PO Box 26901, Oklahoma City, OK 73190, USA; 2 Department of Physiology, College of Medicine, Oklahoma University Health Sciences Center, PO Box 26901, Oklahoma City, OK 73190, USA; 3 Department of Biochemistry and Molecular Biology, College of Medicine, Oklahoma University Health Sciences Center, PO Box 26901, Oklahoma City, OK 73190, USA; 4 Department of Pharmaceutical Sciences, College of Pharmacy, Oklahoma University Health Sciences Center, PO Box 26901, Oklahoma City, OK 73190, USA; 5 Department of Environmental Health Sciences, College of Public Health, Oklahoma University Health Sciences Center, PO Box 26901, Oklahoma City, OK 73190, USA; 6 Microarray Core Facility, Oklahoma Medical Research Foundation, Oklahoma City, OK 73104, USA

## Abstract

**Background:**

To better understand the response of urinary epithelial (urothelial) cells to *Enterococcus faecalis*, a uropathogen that exhibits resistance to multiple antibiotics, a genome-wide scan of gene expression was obtained as a time series from urothelial cells growing as a layered 3-dimensional culture similar to normal urothelium. We herein describe a novel means of analysis that is based on deconvolution of gene variability into technical and biological components.

**Results:**

Analysis of the expression of 21,521 genes from 30 minutes to 10 hours post infection, showed 9553 genes were expressed 3 standard deviations (SD) above the system zero-point noise in at least 1 time point. The asymmetric distribution of relative variances of the expressed genes was deconvoluted into technical variation (with a 6.5% relative SD) and biological variation components (>3 SD above the mode technical variability). These 1409 hypervariable (HV) genes encapsulated the effect of infection on gene expression. Pathway analysis of the HV genes revealed an orchestrated response to infection in which early events included initiation of immune response, cytoskeletal rearrangement and cell signaling followed at the end by apoptosis and shutting down cell metabolism. The number of poorly annotated genes in the earliest time points suggests heretofore unknown processes likely also are involved.

**Conclusion:**

*Enterococcus *infection produced an orchestrated response by the host cells involving several pathways and transcription factors that potentially drive these pathways. The early time points potentially identify novel targets for enhancing the host response. These approaches combine rigorous statistical principles with a biological context and are readily applied by biologists.

## Background

Epithelia are the first line of defense against bacterial infection. Far from being only a passive barrier, epithelia mount a complex and sophisticated response to bacterial infection [1] that can even include synthesis of anti-bacterial peptides [2]. However, the pathogens also can exploit host cell function [3]. In the case of the bladder, the highly specialized and multifunctional urothelium functions remarkably well to protect the host from urinary tract infections. Urothelial cells form a physical barrier against bacterial infection, trap bacteria in mucin and can desquamate, carrying the bacteria out of the bladder [4]. Urothelial cells mount their own immune response against pathogens [5]. Although the response of the urothelium to *Escherichia coli *has been investigated extensively at the molecular level [6,7], the response to gram positive organisms such as *Enterococcus *is poorly understood.

*Enterococcus faecalis *infection of the urinary tract and other organs represents a growing problem. Nosocomial infections with antibiotic-resistant strains are often fatal to hospitalized patients with compromised immune systems [8]. The *Enterococcus faecalis *genome contains a number of antibiotic resistance genes, which leads to rapid selection for resistant strains from a population in the presence of antibiotics and results in difficulty in treating infections by *Enterococcus *[9,10]. Relatively little is known about the defenses elicited by Gram positive organisms such as *Enterococcus*. It is likely that some different mechanisms are involved because *Enterococci *lack the same kinds of fimbriae or pili that are important to the pathogenicity of gram negative uropathogens such as *E. coli *[11,12].

Most studies of host cell response to infection are based on a snapshot determined at a single time point with cells grown on plastic. Cells grown on plastic are a poor model for cells *in vivo*, and determining the time course will not only define the earliest events but can establish the temporal relationships of the response [1]. In this study we followed the transcriptome of urothelial cells growing as a multi-layer urothelial mimetic presented with an overwhelming infection with *Enterococcus faecalis *in order to map out the pathways and genes involved in response to infection at a system level. This study used novel models and bioinformatic approaches to build a system-level roadmap detailing the transcriptome-level response over time. The bioinformatics analysis used F-statistics to first compare expression against system zero-point noise to facilitate identification of expressed genes, then to identify a set of HV genes showing excess variability over that shown by genes only varying for technical reasons. These HV genes encapsulate the response to infection. Clustering and identification of common properties such as pathways and common transcriptional regulatory elements (TREs) developed an integrated picture of the host cell response over time. The urothelial mimetic model duplicates features of infection seen *in vivo *but not in models of cells grown on plastic because the mimetic captures the cell-cell interactions between cells first attacked by the bacteria and cells responding indirectly to the attack. Following the time course has illuminated an orchestrated and integrated response of the cells to infection and the analysis presents an approach accessible to most biologists for developing a systems-level description of the biological events involved with infection. Learning how these defenses function and whether they are different from those elicited by *E. coli *could identify novel means to treat uropathogenic infections by *Enterococcus *in an age of increasing antibiotic resistance.

## Results

### Cell death occurs between 10 and 24 hours post infection

Examination of cell layers infected with bacteria showed that little cell death occurred until 10 hours post infection, at which time cells began to die in significant numbers. Cells at the top in contact with the bacteria died first. Confocal images are shown in the supplemental material (Additional file 1, image A). By 12 hours about 50% of cells had died and by 24 hours post infection, virtually all the cells were dead. Also shown in the supplemental material (Additional file 1, image B) is a confocal image of bacteria on the urothelial layer. The bacteria remained attached to the apical surface of the layer and no evidence for entry into cells was seen. Thus, the gene expression response of the urothelial mimetic will represent both the response of the cells in immediate contact with the bacteria as well as cells that are connected to the outer layer and which respond to signals from those cells that are infected.

### Determination of system-level noise, normalization and identification of hypervariable (HV) and very hypervariable (VHV) genes

**Figure 1:**
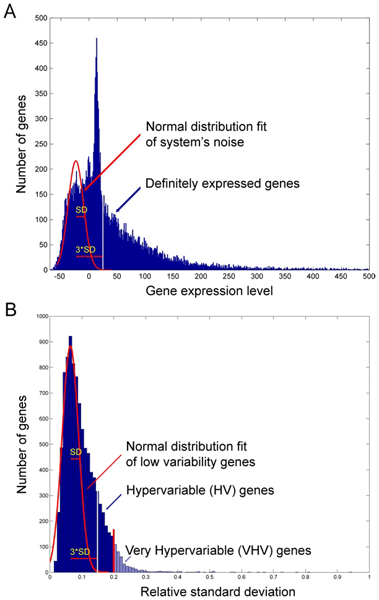
**Histogram of relative standard deviation distribution of 9,553 expressed genes**. The normal distribution of low-variable genes determined from the left portion of the histogram is superimposed on the actual histogram. SD – standard deviation for the normal distribution, 3*SD (white vertical line) – cut-off level for HV genes, red vertical line – cut-off for VHV genes with relative SD ≥ 0.2.

The means used to analyze expression data and identify significantly altered gene expression is based upon F-tests against system-level noise. The assumptions behind this approach are briefly presented here along with the results. A fuller description is provided in Methods. This approach allows the entire data set to be used for a global assessment of noise, that is variation arising from random, technical sources, and reduces the need for replicates. Variation in expression in excess of this technical variation is assumed to arise from biological mechanisms. Expression data are normalized to the uncertainty in the zero point of expression because this value determines the certainty of stating that a gene is expressed above background. Figure 1A shows the frequency histogram of all the expression values for one array prior to normalization. The bimodal distribution is clearly evident. The leftmost mode is due to technical variation about the zero point and represents probe sequences to which nothing has hybridized, that is the genes are not expressed, whereas those in the rightmost mode represent probes to which binding occurred. The stringency of the threshold for filtering expression expressed is selectable. In this study, a value of 3SD above noise was selected. Of the 21,521 unique genes, 9553 were expressed at more that 3 standard deviations (SD_noise_) above noise in at least one time point.

The frequency histogram of relative standard deviations for the 9553 expressed genes over 10 time points showed a right-side skewed distribution (Figure 1B). The assumption is that this distribution represents the contributions of biological and technical variability and that the magnitude of the technical variability is represented by the left half of the curve, which is Gaussian. The last portion of the frequency histogram of relative standard deviation frequency fitted a normal distribution with a mean of 0.065 and SD_noise _of 0.015. Thus, the microarray is highly reproducible with an average relative standard deviation of only 6.5% and with 90% of measurements being within 9.5% assuming only technical variation. This value agreed closely with the relative standard deviations of probes that were replicated on the array. Using a value of 3 × SD_noise _to identify a threshold above which variation is identified as arising from biological sources, 1409 genes were identified as being hypervariable, that is in expressing variability above that due to technical reasons. The remaining 8142 genes are assumed to express only technical variation and therefore are constant in expression biologically. Reasoning that to identify the underlying pathways that are altered by infection a smaller number of "beacon" genes would suffice and simplify the pathway analysis (which can only construct pathways or networks out of 35 genes), a set of VHV genes was delineated by setting the threshold to 0.2. This is more than 13 times SD_noise_, so the probability that these genes would be identified by chance is p < 0.1129 × 10^-20 ^without correction for multiple comparisons.

### Very Hypervariable (VHV) genes show unique clustering at different time points

**Figure 2:**
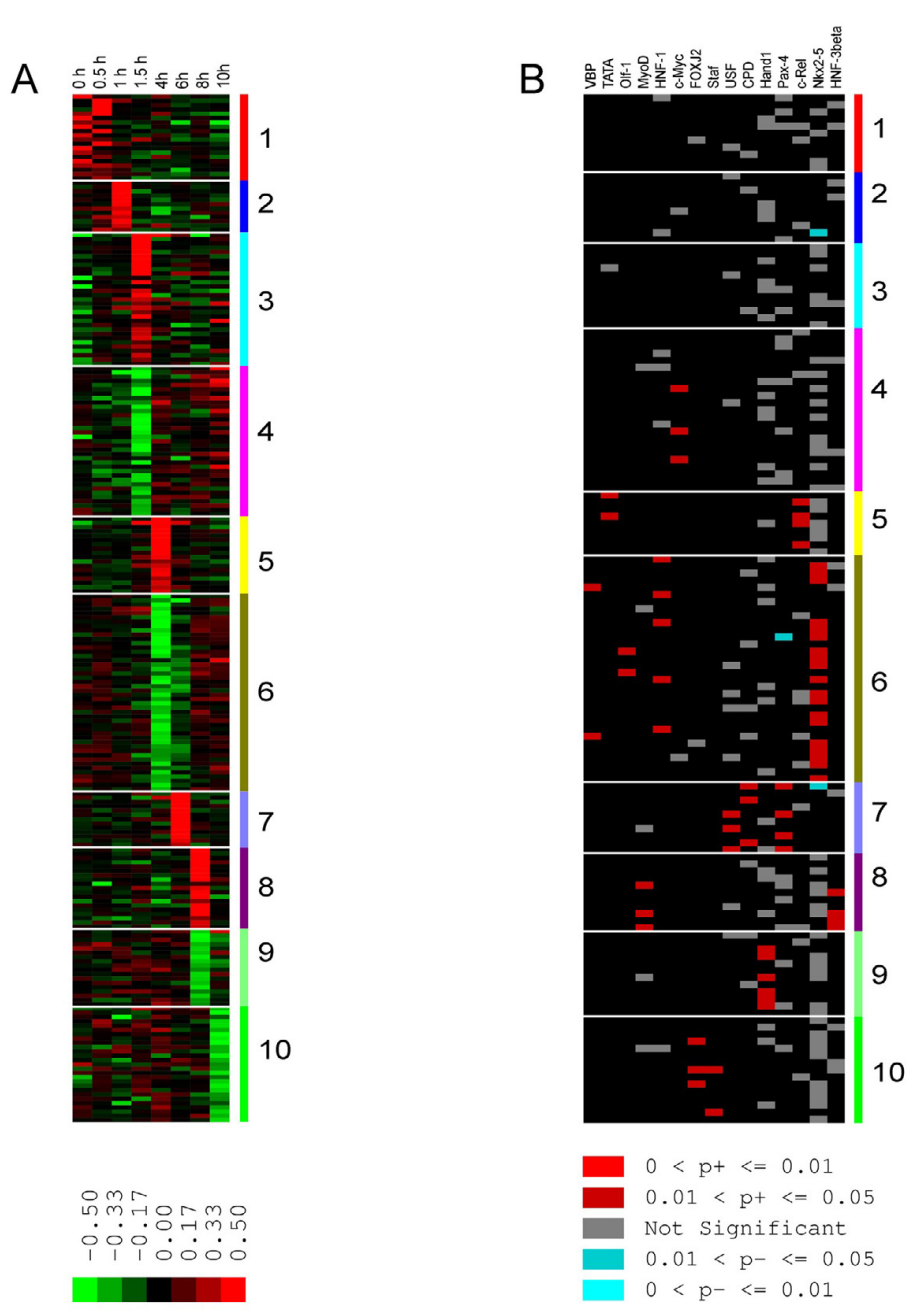
**K-means and PAINT clustering of VHV genes over the time course of infection**. A) Map of up- and down-regulated gene clusters assembled from 239 VHV genes. Red/Green mark increase/decrease in relative gene expression level referenced to the median of the gene over time, respectively. B) Common TREs for given clusters. Color bars at right represent different gene clusters. Red indicates genes sharing overrepresented (p < 0.05) TREs, grey/blue mark genes with not significantly overrepresented/underrepresented TREs, respectively. A larger version complete with gene names is provided in the Additional file 3. A schematic diagram of the significant functions identified by IPA can be found in Figure 5.

**Figure 3:**
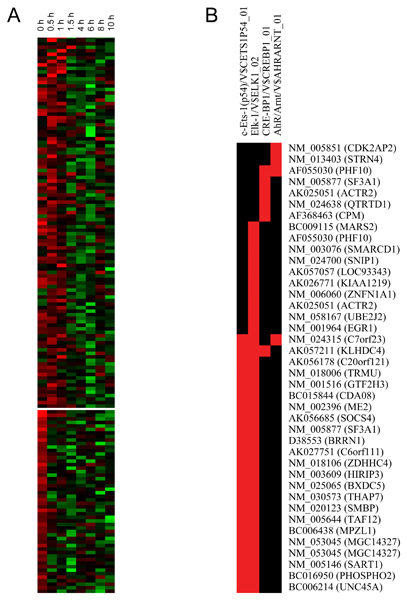
**K-means and PAINT clustering in expanded early response HV genes**. A) K-means clustering of 140 and 52 genes upregulated early and dropped afterwards. B) Common TREs for those genes, filtered by p < 0.05 and FDR < 0.3.

The characteristics of the 239 VHV genes are listed in Additional file 2 sorted by clusters. K-means clustering yielded 10 distinct clusters, with up- or/and down-regulated profiles at different time points (Figure 2A). For an enhanced version of this figure with legible gene names refer to Additional file 3. One result of clustering the VHV genes as opposed to the entire set of HV genes is that the heatmaps are sharpened. The clustering clearly is driven mostly by either an increase or a decrease in expression at a single time point. However, a more familiar pattern is observed when the entire 1409 HV genes are clustered. This is illustrated in Figure 3A, which shows the cluster equivalent to cluster 1 in Fig. 2A, but derived from the complete set of HV genes. Clusters 1 and 2 in Figure 2A are a subset of the cluster shown in Figure 3A, and a more smeared picture of down regulation of expression over the early time points is seen. The clusters can be divided into three major groups by time. The early response group, comprised of cluster 1–3, represented genes whose expression changed during 0–1.5 hours post-infection. The middle response group, containing clusters 4 through 7, represented an intermediate cascade of events during 4–6 hours post infection. The late response group of clusters 8–10 showed responses at 8–10 hours post infection.

Genes with similar expression profiles are assumed to share functional connections. Finding properties other than expression profile shared by a gene cluster increases the confidence that the clustering actually reflects a functional, biological connection. Because co-regulation by common transcriptional regulatory elements (TREs) represent a major mechanism for regulation of gene expression, the finding that particular TREs are statistically over-represented in a gene cluster suggests that the TRE could drive the observed clustering. Figure 2B shows the map of TREs by clusters as identified by the Promoter Analysis and Interaction Network Toolbox (PAINT). While several clusters showed significant over-representation of one or two TREs, which suggests they may play a role in organizing the clusters, other clusters, particularly the early ones, do not show any over-representation of TREs derived from the set of VHV genes. A second means for examining the biological significance of clustering was to determine whether genes forming clusters also could be organized into plausible networks. This analysis was performed with Ingenuity^© ^Pathway Analysis (IPA), a network-identifying tool that is based upon human curated literature.

### Early immune, cytoskeletal and estrogen receptor responses immediately following Enterococcus infection

The early response group represents genes that spiked at early time points (0–1.5 hours post infection) or dropped immediately following the infection. Neither IPA nor PAINT analyses identified any significant ontologies or TREs for the VHV genes (Table 1). However, several pathways were identified by IPA in these very early responses from the VHV genes. Included are early immune cell response (*PTCRA*, *PPIB*), immune response via *IL-2 *(*DGKE*) and *NF-κB *(*H2AFY2*, *PIP5K1A*) pathways. Among the induced cytokine genes was *TNF*, not identified as being hypervariable but present here as a driving force for a network of genes up-regulated 1.5 hours post infection as suggested by IPA. *IL2 *and *TNF *are both hallmarks of *NF-κB *activation. Inhibition of immune response was also suggested. *PIP5K1A *plays role in cyclosporine A-mediated immunosuppression and, as well as *PPIB*, in phosphoinositol activity. Cell cycle regulation (*MAD1L*) and actin-related cytoskeleton remodeling linked to *Rac *(*PIP5K1A*, *ACTR2*, *TEKT1*) were also observed. The networks found for all clusters are shown in Additional files 4, 5, 6, 7, 8, 9, 10, 11, 12, 13, 14, 15. Additional file 16 contains the legends for the IPA generated networks.

**Table 1:** Genes from each cluster assembled in networks and their top functions/canonical pathways. Gene names in bold are "focus genes" identified as VHV and served to identify key elements in hypothetical networks constructed by IPA. Statistically significant top functions and canonical pathways are identified by IPA. Hypothetical networks identified by IPA as being potentially present were pruned to remove genes that were not identified as being expressed.

**Cluster**	**Gene names**	**Network's top functions**	**Canonical pathways**
#1 0.5 hours post infection, up	***ACTR2*, *C12orf23*, *CGI-38*, *CPM*, *DGKE*, *FLJ22471*, *GALNT10*, *H2AFY2*, *LOC197350*, *LRRC51*, *MAD1L1*, *PHOSPHO2*, *PIP5K1A*, *PPIB*, *PTCRA*, *PTPRN2*, *TEKT1*, *TMTC4***, *,* No significant networks assembled	Cell Cycle, Cell Death, Cell-to Cell Signaling and Interaction, Immune and Lymphatic System Development and Function	O-glycan biosynthesis, Phospholipid Degradation, Inositol Phosphate Metabolism, Glycerophospholipid Metabolism, Glycerolipid Metabolism
#2 1 hours post infection, up	*ACP1*, *AKT2*, *BBC3*, *CD19*, *CHGA*, *CKM*, *CSDA*, *CTSD*, *DDX5*, *EEF2*, ***EGR1***, *GTF2I*, *HAS2*, *HOXA5*, *IER3*, *IGF1*, *MYC*, *PGK1*, ***PRDM1***, *PRNP*, ***ROCK2***, *RPS6*, ***SRC***, *TLN1*	Cellular Growth and Proliferation, Cellular Development, Cell Death, Cell-to-Cell Signaling and Interaction, Tissue Development	Neuregulin Signaling, Ephrin Receptor Signaling, Wnt/β-catenin Signaling, PDGF Signaling, Integrin Signaling, ERK/MAPK Signaling, B Cell Receptor Signaling
#3 1.5 hours post infection, up	*ADK*, ***APBB1***, ***BTN3A3***, *CHST2*, *CLIC1*, ***EB-1***, *EI24*, *ENAH*, *F2*, ***FARS2*, *FAS***, *FOS*, ***GNPAT*, *GRB7*, *IL2RA*, *KAL1*, *NDRG1***, *PDRG1*, ***PRKAB2***, *SMARCB1*, ***TCF3***, *TNF*, *TP53*, *TP73L*, *TWIST2*, ***VASP***	Cellular Movement, Organismal Survival, Connective Tissue Development and Function, Cell Cycle	Apoptosis signaling, Wnt/β-catenin Signaling, PPAR Signaling, p38 MAPK Signaling, Integrin Signaling, IL-6,-2,-10 Signaling, Death Receptor Signaling
#4 1.5 hours post infection, down	*AATF*, *AKT3*, ***ARNTL*, *CENPF*, *COL18A1***, *CREG1*, *CRI1*, ***CTDSPL***, *E2F1*, *E4F1*, *EP300*, ***FEZ1***, *HIF1AN*, ***KCNJ1***, *KLF5*, ***POLR2F*, *PPT2***, *PRKCZ*, *RB1*, *RBBP9*, ***SLC29A2***, *SRC*, ***STAT5B***, *TBP*, *UMPS*, ***ZBTB16***	Cellular Growth and Proliferation, Gene Expression, Cellular Development, Cell Death, Cell Cycle	Estrogen Receptor Signaling, NF-κB Signaling, Neuregulin Signaling, Xenobiotic Metabolism Signaling, Wnt/β-catenin Signaling, Pyrimidine Metabolism, PTEN Signaling, PPAR Signaling, Jak/Stat Signaling, Integrin Signaling, IL-2 Signaling
#5 4 hours post infection, up	***ABCA1***, *ALPP*, *C7*, *CCNG2*, *ENPP1*, ***ERBB3***, *F3*, *FLOT1*, *FPRL1*, *G6PD*, *IL1B*, *KLF10*, ***KRT17***, *MEFV*, *NAB2*, *PCSK1*, ***PTX3*, *RRS1***, *S100A6*, *SERPINH1*, *SLC20A1*, *TGFB1*, *THBD*, *TNFAIP2*	Cellular Growth and Proliferation, Hematological Disease, Cellular Movement, Organismal Development, Cell-to Cell Signaling and Interaction	Complement and Coagulation Cascades, p38 MAPK Signaling, Xenobiotic Metabolism Signaling, Wnt/β-catenin Signaling, TGF-β Signaling, Starch and Sucrose Metabolism, Riboflavin Metabolism, Purine Metabolism, PPAR Signaling
#6 4 hours post infection, down	***ARL3***, *CASP14*, ***FCER1A***, *FOXC1*, *G0S2*, *HOXA9*, *KIF3C*, *KPNA3*,***MLL***, *NDUFS4*, ***NDUFV2*, *PEX14*, *RANBP9*, *RBL1***, *RFP*, *SERPINB10*, ***SERPINC1*, *SERPINE1*, *SLC34A1***, *SLC7A1*, ***SMYD3***, *TFE3*, *TGFB1*, *TNF*, *WWOX*	Cellular Development, Cellular Growth and Proliferation, Hematological System Development and Function, Immune and Lymphatic System Development and Function	Ubiquinone Biosynthesis, TGF-β Signaling, p38 MAPK Signaling, Oxidative Phosphorylation, Complement and Coagulation Cascades, Cell Cycle G1/S Checkpoint Regulation, Xenobiotic Metabolism Signaling, Wnt/β-catenin Signaling, PPAR Signaling
#7 6 hours post infection, up	*P29*, *CIRH1A*, *KIF1B*, *GPR51*, *GPS1*, *BMPR2*, THBD*,* No significant networks assembled	Cell Morphology, Cardiovascular Disease, Genetic Disorder, Nervous System Development and Function, Organ Morphology	Pentose Phosphate Pathway, EGF Signaling, IL-2 Signaling, Jak/Stat Signaling, Fructose and Mannose Metabolism, Galactose Metabolism
#8 8 hours post infection, up	***CCR2***, *CDC45L*, *CRABP2*, ***CSF3R***, *DIO1*, *E2F1*, ***EXOSC9***, *GBP2*, *IL4*, *IL6*, *INDO*, ***KCNJ5***, *KIAA0101*, *MAZ*, ***NR1H4*, *NUP62*, *PFKL***, *POLD1*, *PROS1*, ***RAF1***, *RXRA*, *SP1*, ***THRA***	Gene Expression, Cell Death, Hepatic System Disease, Cellular Function and Maintenance, Cellular Growth and Proliferation	Xenobiotic Metabolism Signaling, IL-10,-6 Signaling, PPAR Signaling, VEGF Signaling, Tryptophan Metabolism, TGF-β Signaling, T Cell Receptor Signaling, Pyrimidine Metabolism
#9 8 hours post infection, down	*ALAD*, *CPT2*, *CPT1A*, *CRYAB*, *EEF2*, *GAS1*, ***GCK***, *GSK3A*, *INSR*, *MNT*, *MXI1*, *MYC*, *NDRG1*, ***ONECUT1***, *PDE3B*, *PKLR*, *PTPN2*, ***PTPRG***, ***RPL5***, *SOCS6*, *SORBS1*, *TAT*, *TUB*	Metabolic Disease, Cell Cycle, Connective Tissue Development and Function, Cellular Growth and Proliferation, Carbohydrate Metabolism	Insulin Receptor Signaling, Wnt/β-catenin Signaling, Purine Metabolism, Glycolysis/Glyconeogenesis, Fatty Acid Metabolism, Tyrosine Metabolism
#10 10 hours post infection, down	*ACTN4*, *ADAM10*, *AIM1*, ***ARID1B***, *COL2A1*, *CTNNB1*, ***CTNNBIP1***, *CTSH*, *FZD8*, *IHH*, ***LFNG***, ***MMP2***, *NOTCH1*, *NRP2*, ***PIN1***, ***SFTPB***, *SLC26A2*, *SMARCA4*, ***SOCS1, STXBP3***	Organismal Development, Embryonic Development, Tissue Development, Cellular Development	Wnt/β-catenin Signaling, NOTCH Signaling, VEGF Signaling, Purine Metabolism, PI3K/AKT Signaling, Jak/Stat Signaling, Interferon Signaling, Integrin Signaling, IL-6,-4,-2 Signaling

Because so many of the VHV genes in the early time points were poorly annotated, our assumption that the statistically valid and biologically relevant networks could be identified from the ontologies and TREs of the VHV genes was questionable for the early time points. Moreover, the statistical validity of canonical pathway inclusion was weakened as well. We therefore clustered all 1409 HV genes and identified 192 genes up-regulated at early time points and down-regulated afterwards. A full list of the 192 HV genes in cluster 1 can be found in Additional file 17. Clustering of this expanded set of early response genes is shown in Figure 3A. Out of 192 genes 86 were annotated sufficiently for generating networks. Six networks showed significant overrepresentation of cellular growth and proliferation, immunologic response ontologies, as listed in Additional file 18. Additional canonical pathways emerged as well as additional members in pathways identified from the VHV genes alone. Four genes (*GTF2H3*, *NR0B1*, *POLR2K*, *TAF12*) were significantly overrepresented in the estrogen receptor signaling canonical pathway. PAINT analysis identified four significantly overrepresented TREs (*c-Ets-1(p54)*, *Elk-1*, *CRE-BP1*, *AhR/ARNT*) among this expanded set of genes, as shown in Figure 3B. Of these transcription factor genes only *CRE-BP1 *was not expressed above noise level. The higher resolution view offered by the expanded HV gene set as well as identification of *Elk-1 *and *Ets-1 *transcription factors support the identification of immune response as an early event.

### Dysregulated signaling during the middle time period

While up-regulation of gene expression characterized the early response, the intermediate response (middle response group) contained both up-and down-regulated genes at different time points (Figure 2A). Cluster 2 contained genes up-regulated at 1 hour post infection. IPA assembled one significant ontology with cellular growth and proliferation as the top function. Genes in this cluster are overrepresented in chemokine signaling (*ROCK2*, *SRC*), various complex events, from growth, differentiation (*EGR1*), membrane trafficking and phosphatidylinositol (*PI*) activity (*ZFYVE1*) to immune response via interleukin pathways (*PRDM1*, *TRIM5*). *SRC *and *EGR1 *regulate *MYC *transcription factor (TF), which played an essential role in the subsequent time period. Part of the *NF-κB *signaling pathway is still seen at this time point where *IKIP *(*IKK *interacting protein) is up-regulated. Protein ubiquitination genes (*TRIM5*, *UBE2J2*) were also noted.

At 1.5 hours post infection time point there are two sets of genes, clusters 3 and 4 containing up- and down-regulated genes, respectively. While no significant transcription factors could be identified driving genes in cluster 3, IPA built one network with genes responsible for cell death (*FAS*, *IL2RA*, *TCF3*), cell cycle progression/arrest (*TCF3*, *APBB1*) (Table 1). Most of the genes in this cluster are regulated by various interleukin signaling pathways. Down-regulated genes in cluster 4 were driven by the *c-MYC *transcription factor, and the majority were assembled into one network with cell cycle, cancer and cell morphology as the top functions. Genes labeled by IPA as being cancer related (*COL18A1*, *STAT5B*, *CTDSPL*) were also involved with infection as were genes involved in apoptosis and growth (*CTDSPL*, *POLR2F*, *ZBTB16*). Solute carrier family genes (*SLC29A2*, *SLC35B3*) were down-regulated at this and the subsequent time points. The high prevalence of various transcription factors at this time point should be noted (*ARNTL*, *E2F1*, *TCF3*, *STAT5B*, *ZBTB16*). Additionally, most of the genes identified by IPA as key players, such as *FOS*, *EP300*, *RB1*, *TP53*, *TP73L*, interacting with "beacon" genes were, in fact, expressed but not VHV, thereby supporting the networks assembled by IPA.

Clusters 5 and 6 represented genes up- and down-regulated at 4 hours post infection. Up-regulation of carbohydrate metabolism and small molecule biochemistry were the top functions of the network in cluster 5 while down-regulation of amino acid metabolism, molecular transport and small molecule biochemistry represented two networks in cluster 6 (Table 1). While several genes in cluster 5 are responsive to various interleukins (*ABCA1*, *PTX3*, *RRS1*, *ERBB3*) they also are involved in ATP cycle and lipid metabolism. Several genes (*ABCA1*, *ERBB3*, *PTX3*, *GRP132*) are also involved in cell morphology (cell spreading, transformation) and cellular assembly and organization. Down-regulated genes in two networks in cluster 6 relate to ATP-ADP-GTP cycle (*ARL3*, *AARS*, *RANBP1*, *EEF1A1*, *DPM1 *etc.), apoptosis and cell cycle regulation (*RBL1*, *RAF1*, *MLL*, *WWOX*, *SERPINC1*, *SERPINE1*, *GOS2*, *TMPO*, *TNC*). Down-regulated *SERPINC1* and *SERPINE1 *were parts of interleukin signaling, the former regulated *IL6 *and the latter regulated by *IL1B*. Another solute carrier family of genes (*SLC34A1*, *SLC7A5*) was down-regulated. The network forms a convincing picture organizing different subcellular locations including endoplasmic reticulum and the Golgi apparatus with functions such as protein glycosylation (*DPM1*, *SLC34A1*, *MGAT2*, *ST3GAL1*). Likewise, signaling through several transcription/translation regulators (*MLL*, *EEF1A1*, *SHANK3*) was identified. Interestingly transcription factors identified as over-represented TREs, namely *c-Rel *(cluster 5) and *NKX2-5*, *HNF1 *(cluster 6), were found to be expressed (but not VHV) genes, thereby providing plausibility to the system level analysis. While cluster 7 showed a set of genes involved in cell cycle (*P29*, *APBB2*, *GPS1*) and the *TGFβ*-pathway (*BMPR2*, *THBD*) up-regulated 6 hours post infection, no concise network or significant functions/pathways could be identified (Table 1).

### Gradual decline of cell functions at later time points

Clusters 8 and 9, up- and down-regulated 8 hours post infection, respectively (Figure 2A), represent a variety of functions. Up-regulated genes in cluster 8 were bound in one network with cell morphology, cell death/injury/abnormalities and lipid metabolism as the top ontologies (Table 1). Those genes significantly represented *EGF*- and *IL-2 *signaling pathways. Several genes represented G-protein-coupled and ion receptors (*KCNJ5*, *NR1H4*, *ATP6V1D*). Genes in this cluster expressed *MYOD *and *HNF3B *as over-represented TREs. Down-regulated genes in cluster 9 shared *HAND1 *as an over-represented TRE and were bound in one network with ontologies similar to cluster 5, 6 – carbohydrate/lipid/nucleic- & amino acids metabolism, small molecule biochemistry (Table 1).

The last time point, 10 hours post infection, showed one network of down-regulated genes in cluster 10 related to cancer, carbohydrate metabolism, cell cycle and morphology ontologies. Those genes were significantly overrepresented in the following canonical pathways: interferon/NOTCH/Interleukins/JAK/STAT signaling (Table 1). Degradation processes, such as matrix breakdown, represented by *COL2A1*, *STXBP3*, *ARID1B*, *MMP2*, *CTNNBIP1 *genes. Two zinc finger proteins (*ZNF406*, *ZNF444*) were also down-regulated. Various inflammation- and cell growth/proliferation related pathways represented by *SFTPB*, *SOCS1 *(JAK/STAT cascade), *COL2A1*, *PIN1 *genes also were identified.

## Discussion

For the first time, the response of urothelial cells growing in a urothelial mimetic and presented with an overwhelming *Enterococcus *infection was examined at the level of gene expression from the earliest events until cell death began to overwhelm the cells. The time course illuminated a progressive and orchestrated response to bacterial infection by the urothelial cells. At the earliest time points, the evidence suggests the cells initiate an immune response, cytoskeletal rearrangement and estrogen receptor signaling. Numerous poorly annotated genes identified in the early time period suggest currently unknown functions may be involved as well. The intermediate time points from 4 to 8 hours were characterized by modulation of cellular pathways that were under cellular control but were initiated by the earliest response to *Enterococcus*. In the final time points, the cells were initiating death programs and shutting down essential life processes.

Several characteristics of this model and of transcriptomics in general led us to use a novel systems biology approach to interpreting the data. First, because recent work showing that signaling represents a highly interactive cellular network [13], and even challenges the concept of "pathways", key functional events might only be observed indirectly in the transcriptome. Thus, the usual statistical analysis of finding a few highly differentially expressed genes is likely to be overly simplistic and inaccurate in the absence of an expensive number of replicates. Second, transcripts were derived both from cells that were in direct contact with bacteria as well as from cells whose contact with bacteria was indirect and through cell-cell communication. While the top cell layer in contact with bacteria may produce a range of responses and die quickly, cells underneath may proliferate and respond first to the cells above them and then to bacteria at later time points. This is a feature of natural infection that is captured in the model used in this paper, but the result could be to smear out and obscure the response. Third, most microarray results tend to over-represent high expression genes over those that are expressed near the background, even though the low-abundance transcripts may represent important regulatory genes such as transcription factors. Fourth, with over 21,000 different genes being represented on the array and 10 time points, the resulting data set consists of over 200,000 data points, and determining whether patterns can occur by chance represents a fundamental challenge. We therefore used a very conservative approach such that the probability of any of the "beacon" VHV genes being identified by chance was vanishingly small.

Because transcriptomics data are almost universally underdetermined, there is no single solution to any data set, and, in fact, many solutions are possible. The approach we describe here is based upon differences in variance that are due to technical and biological factors and to the characteristics of microarray experiments in general. The advantage of a variance-based approach is that it is much less dependent upon replicates than are methods based upon comparisons of means. The entire experiment is a replicate for those 8142 genes for which the technical variability exceeds that due to biological factors ("constant" genes), and their distribution of variance allows estimates of the probability of the change in expression of any single gene falling outside specified limits to be made. By adjusting the thresholds of significance, the number of "interesting" genes can be managed, either to identify a large set encapsulating the system variance or a small set of "beacons" that point to the pathways in which they function. By referencing system noise in both determination of expression and identification of "interesting" genes, the signal to noise ratio can be optimized to produce an interpretable picture of the system as a whole. VHV genes serve as these beacons. A key to this approach is that the analysis of array data is linked to network tools such as PAINT and IPA and does not proceed in isolation. That most of the genes suggested by IPA as forming networks with the beacon VHV or HV genes actually were expressed validates this approach and argues against chance variation in selection of VHV or HV genes as being significant.

Time course data are complex because individual time points are related to each other. Given the lack of replicates, methods based on comparisons of means are not applicable, and including 3–5 replicates of each time point makes experiments very expensive. Thus, variance-based analyses have an advantage in analyzing data sets such as this one here. A recently described method [14] compares the goodness of fit of the expression values of each gene over time fitted to a curve or to a flat line. Whether our and that method would yield different results is not clear. Most likely, some genes would be identified as significant in both approaches and some only in one. Whether a markedly different picture of pathways and networks would result is doubtful.

The unsupervised clustering organizes the data based on the observed expression pattern whereas the pathway tool examines the genes in the clusters in light of what is known in the literature. Often, as in the present case little literature about the function of genes in urothelium is available, and therefore functions are inferred from other contexts (T-cell development, for example, in the case of *PTCRA *discussed below). However, the observed expression data represent the functioning of these genes in the context of urothelium responding to *Enterococcus*, and it may be that genes function differently in different contexts. In some cases so little is known about the function of a particular gene and the interaction partners of its protein product that Ingenuity doesn't even recognize it. Thus, failure of a gene to be recognized as fitting into a network may simply reflect lack of information about the function of the gene within the current context or any context (e.g. the open reading frame). Therefore genes that do not fit into networks should not be discarded arbitrarily as "false positives," they could be considered as representing completely novel findings. Thus, these networks represent only first approximations of the actual networks that represent the underlying biology. Inevitably the question of confirmation arises, but this is a far more complex question than is generally considered [15]. Given the generally high correlation between different array platforms and PCR, the value of confirming RNA levels by PCR is limited [16]. Ultimately, the only real confirmation is at the functional protein level in hypothesis-driven experiments. The purpose of the system-level examination of complex processes is to generate hypotheses by identifying previously unrecognized connections among genes and suggesting how genes might function as a system rather than as isolated genes or even canonical pathways. Unfortunately, further investigation is often hampered by a lack of suitable reagents.

During the early period post infection, most of the VHV beacon genes were up-regulated with the exception of one small cluster of genes that was down-regulated 1.5 hours post-infection and a few genes that were expressed at high levels in the control and immediately down-regulated. None of the first three clusters showed any common transcriptional regulatory elements or convincingly suggested ontologies. The higher resolution picture produced by including the entire set of HV genes clarified the picture and identified important functions and pathways that are triggered by interaction with the bacteria. *Enterococcus faecalis *expresses 41 proteins that could interact with urothelial cells or their local matrix and thereby alter gene expression [17]. In depth analysis showed two were likely enzymes with catalytic activities toward carbohydrates, including hyaluronan and chondroitin sulfate. Because urothelium expresses a dense network of surface chondroitin sulfate that acts as a protective element for the urothelium [18], inactivation of this layer may be required before infection can proceed. The very early cytoskeleton organization response in the host cells could result directly from interactions with these bacterial proteins.

**Figure 4:**
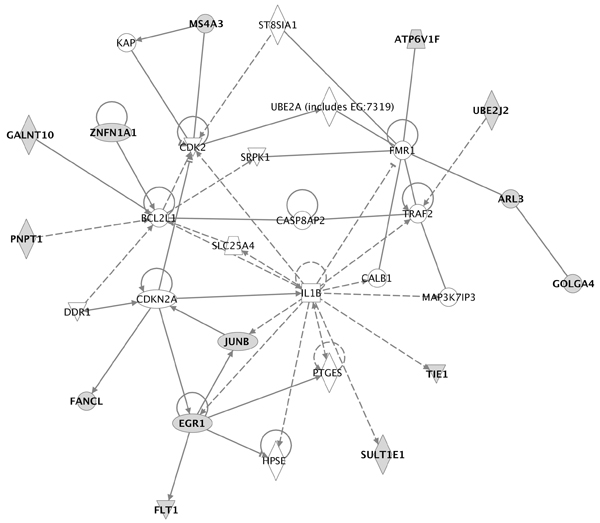
**Example of first plausible network of genes in enhanced cluster 1**. All other networks of interacting genes in this and other clusters are listed in the Additional files 4, 5, 6, 7, 8, 9, 10, 11, 12, 13, 14, 15, 16.

One strongly represented element in the early response genes was initiation of an immune response at the earliest time points. Urothelium, like many epithelia, is capable of mounting an innate immune response [5]. The early immune response is shown clearly by PAINT, which identified the two immune function transcription factors *Elk-1 *and *Ets-1 *from their TREs as well as by identification of a number of genes in immune response networks by IPA; one example is shown in Figure 4. While several of these genes bear names suggesting a specific function in another cell type (e.g. *PTCRA*, or pre T-cell antigen receptor alpha) our data show they are expressed in urothelial cells. Whether they play the same role in urothelial cells as in T-cells respectively remains to be established. Interestingly, four estrogen receptor signaling genes were identified. Estrogen receptor signaling plays a much wider role than the classical estrogen response element signaling [19]. Urothelial cells bear both androgen and estrogen receptors without regard to the sex of the donor [20]. The early responses therefore may represent a fruitful target for interfering with bacterial infection and deserve deeper investigation, particularly because so many of the HV and VHV genes are so poorly annotated. Novel functions and processes are likely to be identified.

The middle time period, 4 to 8 hours post infection represents an active response to infection from endogenous cellular networks. The cells respond by continuing the immune response, up-regulating metabolism, cellular proliferation and development, and cell-cell signaling. Relatively few genes are down-regulated during this period. Two plausible networks were assembled; one network characterized by up-regulation of the focus genes and the other by down-regulation. Interestingly, both include *TGFβ *as an extracellular cytokine. The up-regulated network also contains *TGFβ*, but *IL1B *is present as well, whereas the down-regulated cluster contains *TNF*. Interestingly, the signaling molecules are all expressed at low levels, but consistently above the background. Of considerable interest is the presence of *SERPINC1 *in the down-regulated network. This gene is more familiar as antithrombin III, a regulator of serine proteases in the coagulation cascade in the presence of heparin-like molecules. *SERPINC *is responsible for the puzzling function of "hematologic diseases" in Table 1. Yet, it also is expressed above background. This gene could represent an additional element of protecting the surface glycosaminoglycan layer. *Wnt *signaling also seems to be a target during this time period. This signaling pathway plays a major role in organization of epithelial cells [21]. Inflammatory response pathways driven by *NF-κB *[22-26] and a variety of interleukins [27,28] (*IL2 *in particular) can also be seen early and continuously throughout the timecourse. The *NF-κB *pathway is activated via Toll-like receptors [26,29], especially in bacterial infection [30-32]. We did not observe significant expression of TLRs although some of them (*TLR *6, 7, 8, 10) were expressed close to or slightly above the 3 SD background cut-off. This is in accordance with another study of *Enterococcus *infection [33].

Several transcription factors were implicated by PAINT as controlling individual clusters. In the middle time period, Cluster 4 shows an over-representation of genes containing a TRE for *c-Myc*, and Cluster 5 contains an over-representation of the inflammation-related *c-Rel*. Cluster 6 is enriched in the *Nkx-2-5 *TRE, as well as *HNF-1*, *Olf-1 *and *VBP*. The *Nkx-2-5 *is most likely a regulator of urothelial genes in general [23] and is expressed in all clusters. Cluster 7 shows an over-abundance of genes containing *Pax-4*, *CPD *and *USF *TREs. In the late time points, *HNF-3β*, *MyoD*, *Hand-1*, which also likely is a driver of urothelial genes in general [23], *FoxJ2 *and *Staf *are over-represented. Although PAINT and IPA operate by different principles, nonetheless several of these TREs were implicated by both methods, thereby further supporting their importance. The transcriptional regulatory networks implicated by TRE analysis could help identify networks of genes operating together in bladder that have not been identified to date.

**Figure 5:**
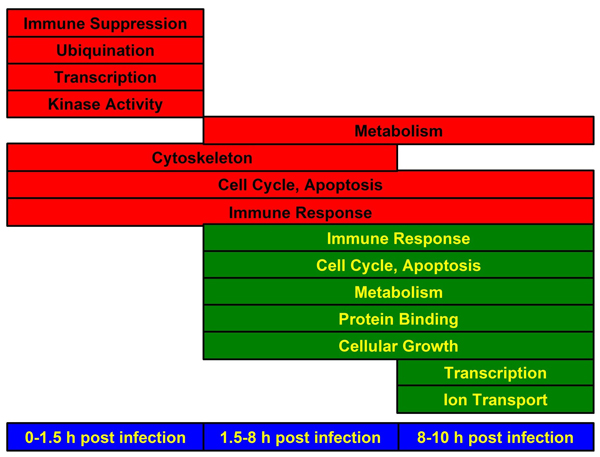
**Schematic diagram of significant functions identified by IPA analysis of clusters shown in Fig. 2**. Functions associated with genes that were up-regulated are shown in red and those that were down-regulated are shown in green.

Connecting clusters across time with IPA suggested some information flow across time from the earliest time points as summarized in Fig. 5. While these comparisons were complicated by the general paucity of detailed annotations of the early response genes, the limited annotations suggest *PTCRA *is linked to *TCF3 *in cluster 3, which is up-regulated 1.5 hours post infection. This points to *Wnt/β-catenin*, which is a transcription cofactor with *TCF/LEF*. Expression of *CTNNB1 *(β-*catenin*), albeit low, tracks the time course of *TCF3 *expression. Other components of this pathway are all either expressed at constant levels or have similar temporal profiles. A second theme is rearrangement of the cytoskeleton as representing an early response, as shown by finding *ACTR2*, *TEKT1*, *CDH24*, and *RPH3AL *as VHV genes. A third theme across time is *Ephrin *signaling. This may be part of a more global picture of cell structural remodeling, where *Wnt/β-catenin *acts along with ephrin/IL2/neuregulin signaling cascades.

The final time period is predominately characterized by down-regulation, with the exception of up-regulation of genes responsible for cell death. Overall the process of degradation can be seen in down-regulation of *Wnt *pathway, metabolism and interleukin signaling concluding cascade of events leading to cell death after *Enterococcus *infection.

While the number of system level studies of host-pathogen interactions currently is small and even smaller when only epithelial cells are considered, the number is growing [1]. However, comparing studies will be complicated by the context problem, and therefore we should perhaps not expect to find much correspondence in individual genes. Common pathways may emerge if they represent core functions (e.g. cell cycle, immune response or apoptosis), and correspondence with previous studies showing these pathways are involved in the host response was observed [1]. Each bacterial system is unique in what it secretes and how it affects the host. Each host cell also has its own unique response system [1]. Until the literature is more comprehensive, comparing studies will be complicated.

Our study for the first time maps the response of human urothelial cells to infection by *Enterococcus *at the transcriptome level from the earliest responses to the initiation of cell death. A novel bioinformatics approach was used that combined rigorous statistical tools in a biological framework to bring out a systems level picture. Although a picture involving reasonably well understood processes could be assembled, the finding that so many early response genes are poorly annotated suggests that the picture of the host cell response is nonetheless very incomplete at the system level. Understanding the early events could yield important clues for preventing infection as well as identify currently unrecognized responses of the cellular network.

## Methods

### Bacterial strains and cell growth

The SV40-immortalized human urothelial cell line HUC-BC(ATCC) was infected with the retrovirus pLEGFP (Clontech, Mountain View, CA) and cells stably expressing green fluorescent protein (GFP) were selected with flow cytometry. Cells infected with the retrovirus pDsRed (Clontech, Mountain View, CA) thus expressing dsRED were used for cross-section visualization. Small intestine submucosa (SIS) membranes encased in plastic inserts (Cook Biotech, West Lafayette, IN) were equilibrated with 3 washes of Hanks Balanced Salt Solution (Invitrogen – Gibco, Carlsbad, CA). After washing, the SIS inserts were incubated in Ham's F12 solution(Invitrogen – Gibco, Carlsbad, CA) with 10% FCS (fetal calf serum) (Invitrogen – Gibco, Carlsbad, CA) for at least 3 hours before cells were placed on membranes. Each SIS insert was placed in an individual well of a 6-well plate (BD Falcon, Bedford, MA) with care taken to ensure air is not captured under the membrane and full contact with the Ham's F-12 media is made. All but 2.5 ml of the media is removed as well as the media on top of the membrane itself. GFP expressing HUC-BC cells were typsinized and brought up in media at a concentration of 150,000 cells/50 μl, and 50 μl seeded onto a membrane surface with area of 50.3 mm^2^. This cell number is sufficient to cover the membrane with a monolayer of cells in a volume sufficient to keep cells within the membrane insert. Cells were grown for 7 days in Ham's F-12 medium containing 10% FCS changed every 2 or 3 days. At day 6, cells were trypsinized from one membrane to obtain a cell number so that the concentration of *Enterococcus faecalis *bacteria would have a multiplicity of infection (MOI) of 10. *Enterococcus faecalis *(strain W32944, UTI isolate) cells from an overnight culture in Brain Heart Infusion were pelleted by centrifugation at 4000 × g for 10 min, washed once with Ham's F-12 medium containing 10% FCS and resuspended in the same medium. For infection, appropriate numbers of cells in 50 μL volume were was layered onto each HUC-BC/SIS membrane and incubations of 0 minutes, 30 minutes, 60 minutes, 90 minutes, 2 hours, 4 hours, 6 hours, 8 hours, 10 hours, and 24 hours were carried out at 37°C in a 5% CO_2 _incubator. Control membranes received 50 μL of Ham's F-12 medium containing 10% FCS.

### Visualization

Two HUC-BC/SIS membranes from each time point within the infection time course were visualized with confocal fluorescence microscopy to determine the time points for microarray analysis. Propidium iodide (Invitrogen – Gibco, Carlsbad, CA) was used to indicate dead cells. Bacteria were labeled with GFP for cross-section visualization in conjunction with HUC cells expressing dsRED.

### RNA isolation and microarray analysis

RNA was isolated at each time point with RNAeasy kit (QIAGEN Inc., Valencia, CA). Cy3 labeled cDNA was synthesized and hybridized onto glass arrays spotted with 22,464 long oligos (~70 mers) from the UniGene database of functionally known genes.

### Data normalization and identification of hypervariable genes

Data were normalized as was described previously [34] using an approach that takes advantage of the statistical and biological properties of microarray experiments to reference expression data and perform classifications and filtering with reference to the variability inherent in the data set as a whole. Removal of duplicate, "Blank" and "Control" values yielded 21,521 unique genes. In order to facilitate identification of genes that are expressed a certain level above background, expression was normalized to the variability of unhybridized probes as described [35]. Briefly, a frequency histogram of the un-normalized expression values yielded a bimodal, right-skewed curve (Figure 1A). The distribution around the first mode was normally distributed, providing a measure of the variability around zero. The expression of all genes was then normalized to this value after subtraction of the zero point. The arrays were then adjusted to each other by robust linear regression, which assumes that the expression of most genes is not altered in the experiment and down-weights the effect on global expression of those that do change. The data set was then filtered to remove all genes showing less that a value of 3.0 for expression. This is equivalent to setting a threshold of 3 SD above background for deciding that a gene is expressed. While this choice is arbitrary, it means that out of roughly 10,000 expressed genes, about 13–14 would represent false positives at any one time point. By requiring expression at more than one time point for inclusion reduces the probability of falsely scoring a gene as expressed to virtually zero. Next, the technical variability in the system was identified from a frequency histogram of relative standard deviation of all the measurements of each gene (Figure 1B). Given that most genes vary only for technical and not biological reasons [34], the standard deviation determined from the normally distributed portion of this histogram defines the system noise in measurement of individual gene expression values. These genes whose variance is normally distributed provide an internal standard of measurement noise (ISMN) [34]. Genes with variances 3SD above the mean of ISMN express more variability than is expected from the internal consistency of the data. These are selected as hypervariable (HV) genes and comprised a total of 1409 genes. While this number captures the excess variability over system noise, it is inconveniently large. The set of HV genes was further filtered to select only those genes with relative standard deviations greater than 0.2. The probability of any of these genes being selected by chance also is vanishingly small. This produced 239 very hypervariable (VHV) genes used for further analysis. These genes serve as "beacons" with which to identify pathways and networks that are perturbed by the process of infection over the time course. We emphasize that it is not necessary to identify at this point every gene that shows a statistically increased probability of modulation. The expression levels of all 21,521 genes are listed on the public database GEO (Series GSE5988).

### Hierarchical clustering

The expression values of 239 VHV genes were clustered by Cluster 3.0 program [36], the next generation of the Cluster program developed by M. B. Eisen [37]. Genes were median-centered and organized into 10 clusters by K-means clustering with 1000 number of runs. The similarity metric was selected as Correlation (uncentered). The results were visualized with Java TreeView program, also the next generation of TreeView program originally developed by M. B. Eisen.

All 1409 HV genes were clustered by K-means clustering with 1000 runs using the Euclidian similarity metric. Genes up-regulated at early time points that then dropped below noise afterwards were identified manually because genes expressed at only 1 time point would otherwise be filtered.

### Pathway and promoter sequence binding analysis

Biologically relevant networks were assembled from sets of genes in each cluster with IPA, a web-based pathway analysis program [38]. IPA uses a genome-scale biological knowledge base, and generates multiple biological networks with associated ontologies from a list of focus genes, in this case the genes in clusters identified by hierarchical clustering. This analysis enables assembly and visualization of direct and indirect physical and biological interactions among a given gene set (focus genes) and other genes that are inferred from literature reports of connections. Each gene identifier was mapped to its corresponding gene object in the Ingenuity^© ^Pathways Knowledge Base. Genes were not weighted by expression levels, and biological networks were built on this assumption. Each network was assigned a significance score calculated as the negative log of the probability of assembling N genes that form a network from a random sampling of the transcriptome. Networks with scores above 3 are considered significant at p < 0.001. The functional analysis of networks identified the biological functions and/or diseases that are most significant to the genes in the network. Canonical pathways analysis identified the most significant known biological pathways for a given set of genes. These networks are not equivalent to canonical pathways because they are based on literature reports of interactions that can be direct or indirect, which is both their strength and weakness. Because these hypothetical networks infer the presence of genes not in the focus gene list, it is necessary to remove genes that are not expressed. This was done in all our pathway figures (Additional files 4, 5, 6, 7, 8, 9, 10, 11, 12, 13, 14, 15), ontologies and canonical pathways. Thus, every gene that is shown is expressed at least 3 SD above the background noise. These pathways, such as shown in Figure 4 are then designated as "plausible" networks because all the members are expressed at the RNA level, at least.

TREs upstream of the clustered VHV genes were identified using the web-based program PAINT [39,40]. PAINT queries the Transfac™ database and calculates the probability that the TREs identified in a given list of genes differ from a random sample of TREs. In this case, the validity of partitioning into clusters was tested by comparing the TREs found in each cluster against the entire list of VHV genes. This provided a map of TREs significantly overrepresented in a given cluster against a significance threshold of p < 0.05. For more reliable results filtering with FDR < 0.3 criterion was used, when specified. It should be noted that a random collection of genes will not form statistically significant pathways or networks. Neither will they contain over-represented TREs. Thus the finding that a set of genes contain common TREs or fit into known networks supports that they are neither randomly selected by chance or the product of technical error.

## List of abbreviations

ATCC – American Type Culture Collection

FCS – fetal calf serum

GFP – green fluorescent protein

HUC – human urothelial cells

HV – hypervariable

IPA – Ingenuity^© ^Pathways Analysis

ISMN – internal standard of measurement noise

MOI – multiplicity of infection

PAINT – Promoter Analysis and Interaction Network Toolset

PI – phosphatidylinositol

SD – standard deviation

SIS – small intestine submucosa

TF – transcription factor

TRE – transcriptional regulatory element

VHV – very hypervariable ("beacon" genes)

## Competing interests

The authors declare that they have no competing interests.

## Authors' contributions

MD performed the overall analysis of hypervariable genes, prepared figures, tables and additional material and helped to draft and finalize the manuscript. KDK carried out the microarray studies, including isolation and amplification of mRNA. REH wrote the first draft of the manuscript and directed the overall study; RS assisted with the interpretation of the TRE analysis; NS and ASB contributed to the bacterial infection part of the study, including interpretation; MBC directs the microarray facility and supervised the quality of the array studies and their interpretation. All authors read and approved the final manuscript.

## Supplementary Material

Additional file 1 **Confocal microscopy revealing the amount of cell death at different time points**. A) Green – live HUC cells expressing GFP, red – dead HUC cells stained with propidium iodide. B) Cross section of HUC cells expressing dsRED and bacteria expressing GFP on top. Click here for file

Additional file 2 **Full list of 239 VHV genes in different clusters**. GenBank accession numbers, gene names and description provided. Click here for file

Additional file 3 Expanded versions of K-means and PAINT clustering of VHV genes showing gene names and GenBank accession numbers. Click here for file

Additional file 4 **Additional plausible network 2 in cluster 1**. Hypothetical networks identified by Ingenuity^© ^as being potentially present were pruned to remove genes that were not identified as being expressed. Click here for file

Additional file 5 Additional plausible network 3 in cluster 1. Click here for file

Additional file 6 Additional plausible network 4 in cluster 1. Click here for file

Additional file 7 Additional plausible network 5 in cluster 1. Click here for file

Additional file 8 Additional plausible network 6 in cluster 1. Click here for file

Additional file 9 Plausible network for Cluster 2, 1 hour post infection, up-regulated Click here for file

Additional file 10 Plausible network for Cluster 3, 1.5 hours post infection, up-regulated Click here for file

Additional file 11 Plausible network for Cluster 4, 1.5 hours post infection, down-regulated Click here for file

Additional file 12 Plausible network for Cluster 5, 4 hours post infection, up-regulated Click here for file

Additional file 13 Plausible network for Cluster 6, 4 hours post infection, down-regulated Click here for file

Additional file 14 Plausible network for Cluster 8, 8 hours post infection, up-regulated Click here for file

Additional file 15 Plausible network for Cluster 9, 8 hours post infection, down-regulated Click here for file

Additional file 16 Legends for IPA generated networks Click here for file

Additional file 17 **Full list of 192 HV genes in cluster 1**. GenBank accession numbers, gene names and description provided. Click here for file

Additional file 18 **Genes from expanded cluster 1 assembled in networks and their top functions/canonical pathways**. Gene names in bold are "focus genes" identified from clusters formed from VHV genes. Non-expressed genes were manually removed from the networks. Statistically significant top functions and canonical pathways are identified by IPA. Click here for file
