# Supplementary figures and images for: Systems biology approach for mapping the response of human urothelial cells to infection by Enterococcus faecalis
Source: BMC Bioinformatics. 2007 Nov 1;8(Suppl 7):S2. doi: 10.1186/1471-2105-8-S7-S2 (PMC2099488; doi:10.1186/1471-2105-8-S7-S2)

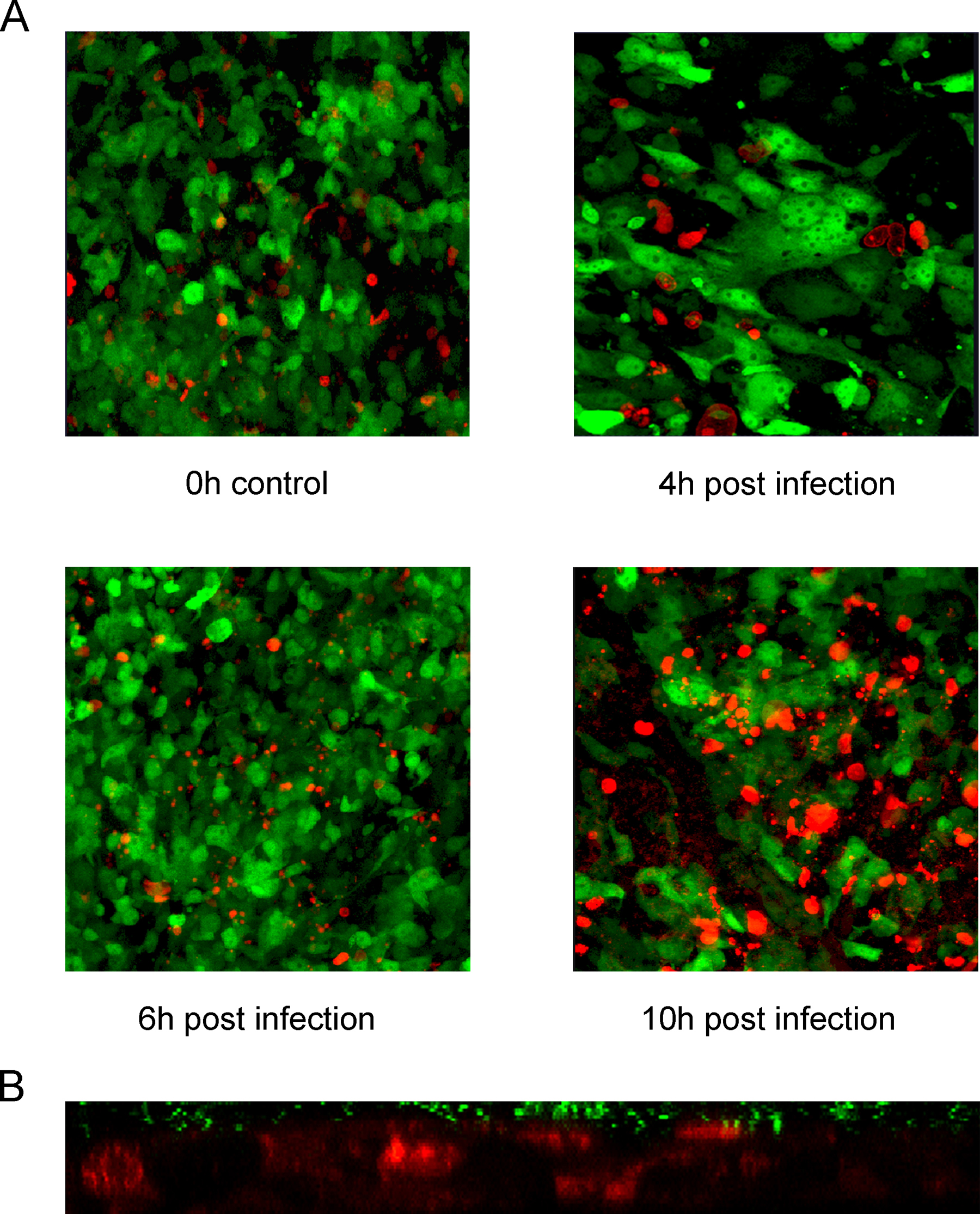

Supplement: Additional file 1 — Confocal microscopy revealing the amount of cell death at different time points. A) Green – live HUC cells expressing GFP, red – dead HUC cells stained with propidium iodide. B) Cross section of HUC cells expressing dsRED and bacteria expressing GFP on top. [file 1471-2105-8-S7-S2-S1.jpg]

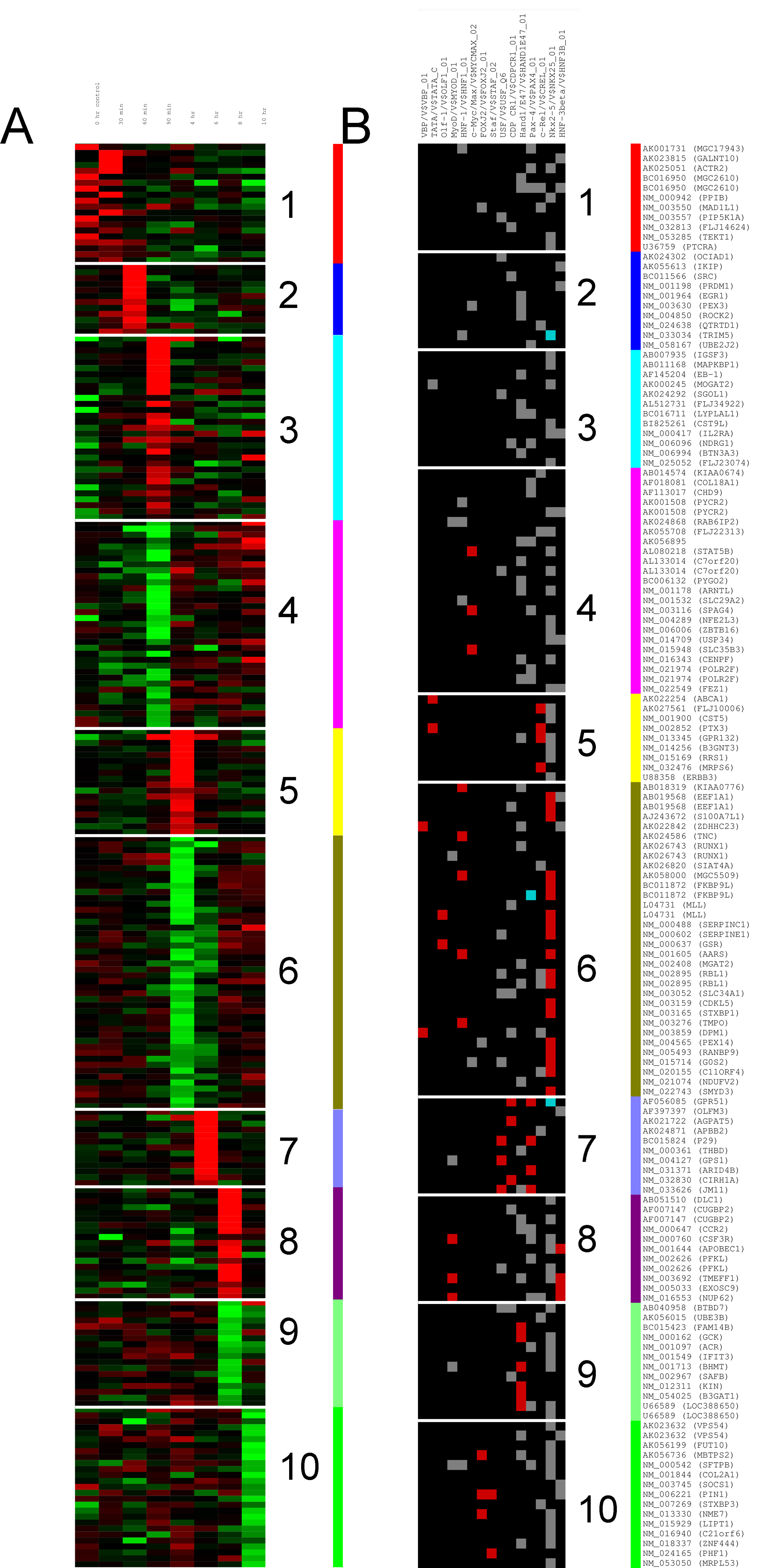

Supplement: Additional file 3 — Expanded versions of K-means and PAINT clustering of VHV genes showing gene names and GenBank accession numbers. [file 1471-2105-8-S7-S2-S3.jpg]

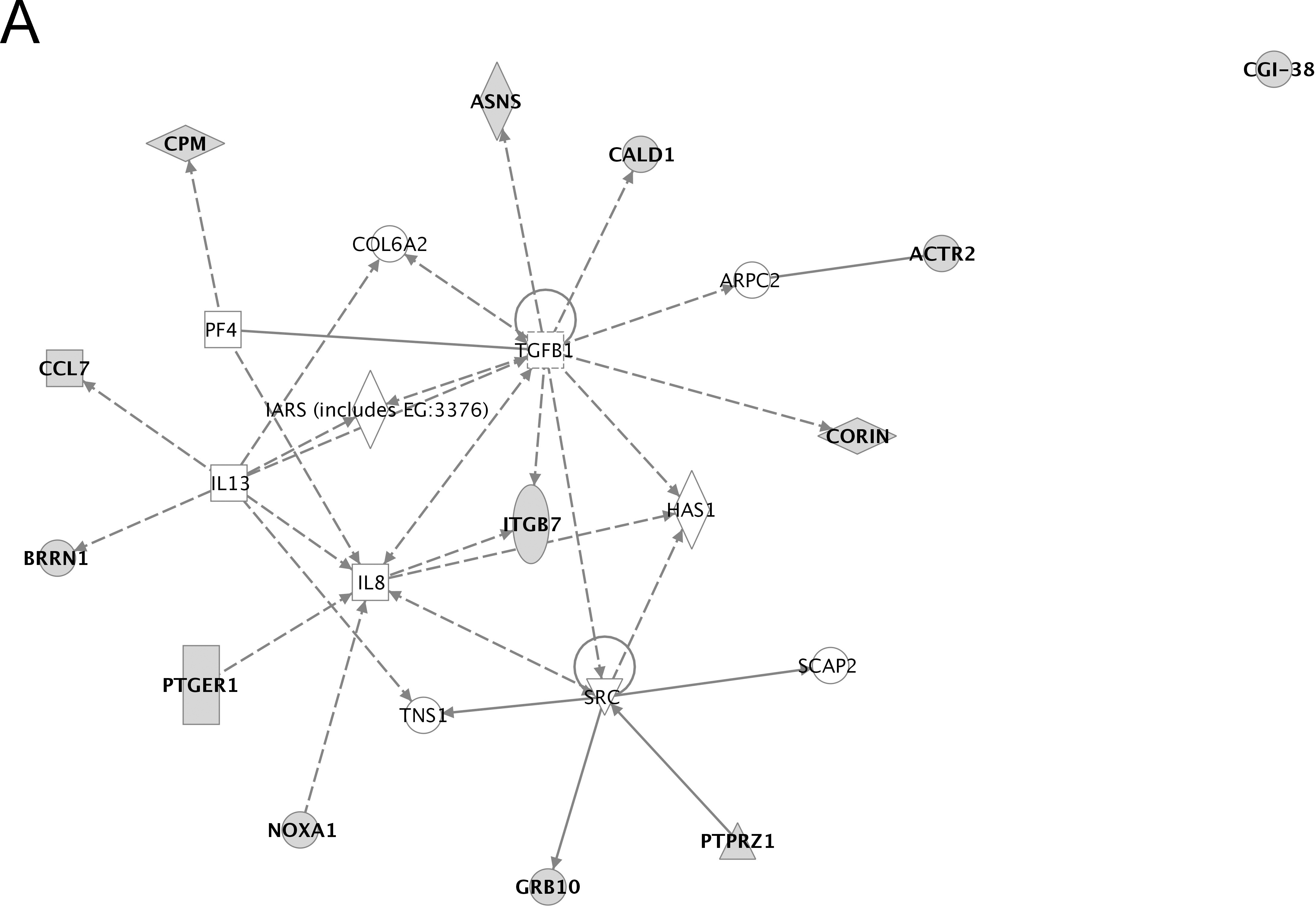

Supplement: Additional file 4 — Additional plausible network 2 in cluster 1. Hypothetical networks identified by Ingenuity© as being potentially present were pruned to remove genes that were not identified as being expressed. [file 1471-2105-8-S7-S2-S4.jpg]

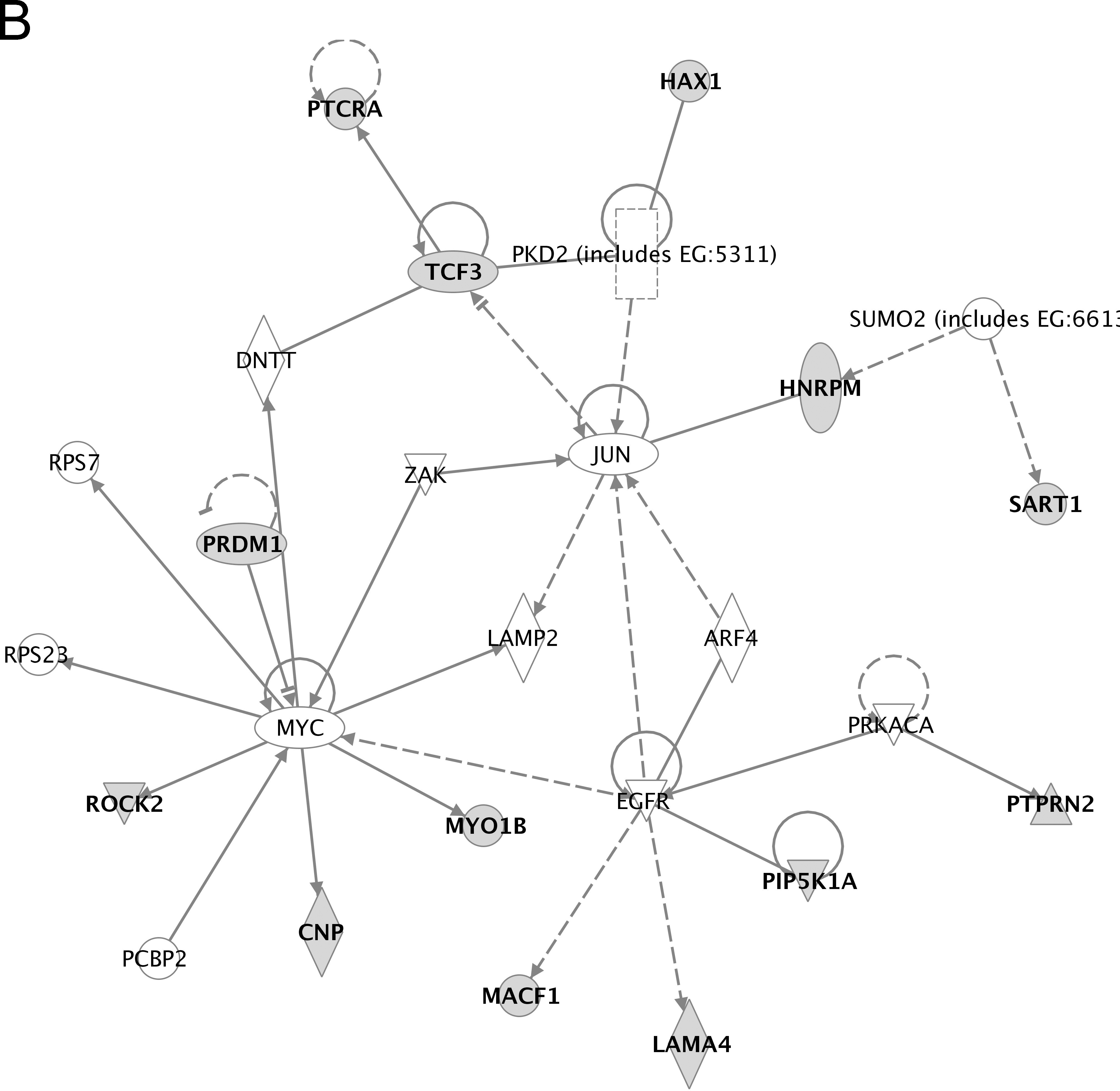

Supplement: Additional file 5 — Additional plausible network 3 in cluster 1. [file 1471-2105-8-S7-S2-S5.jpg]

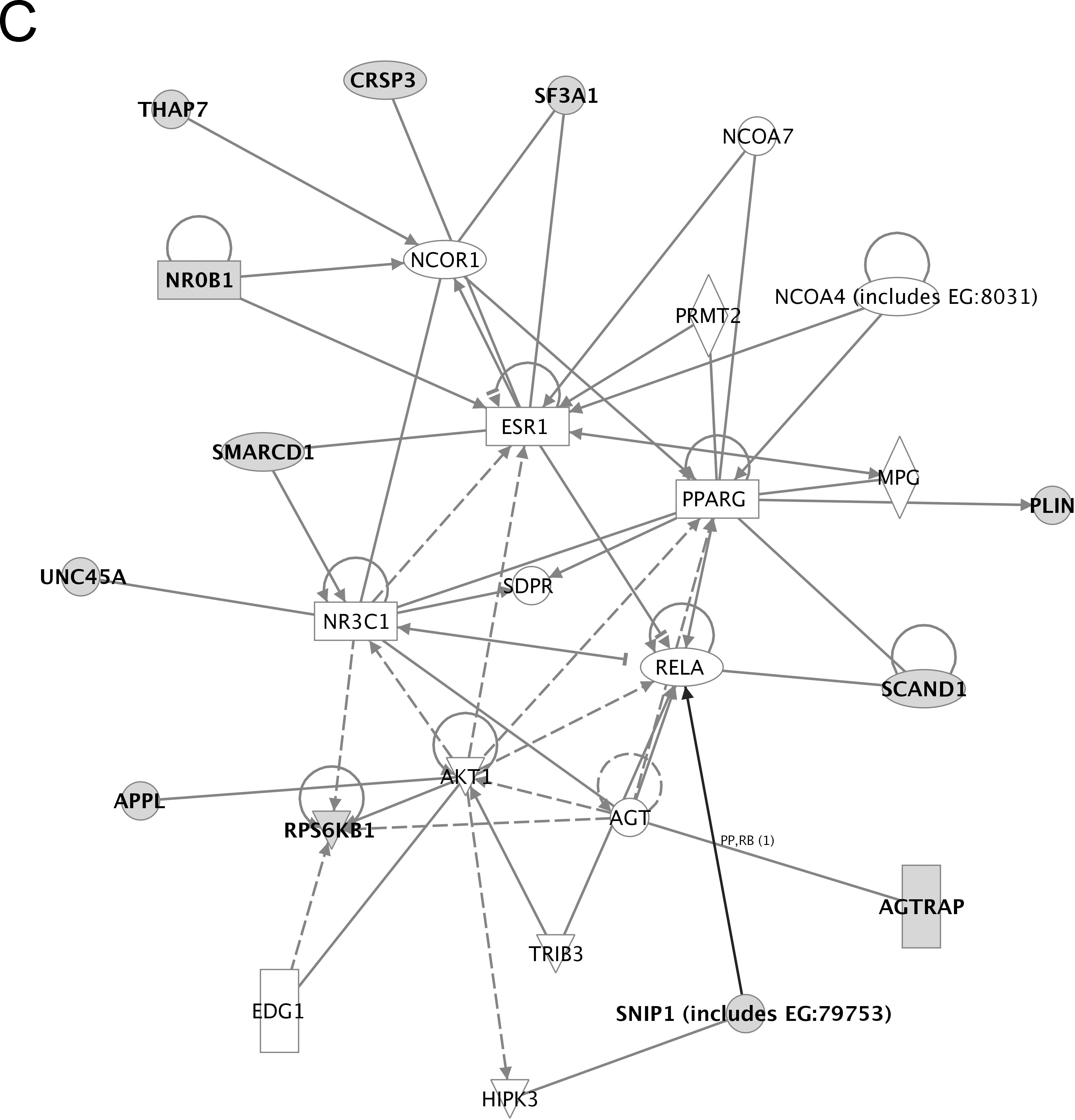

Supplement: Additional file 6 — Additional plausible network 4 in cluster 1. [file 1471-2105-8-S7-S2-S6.jpg]

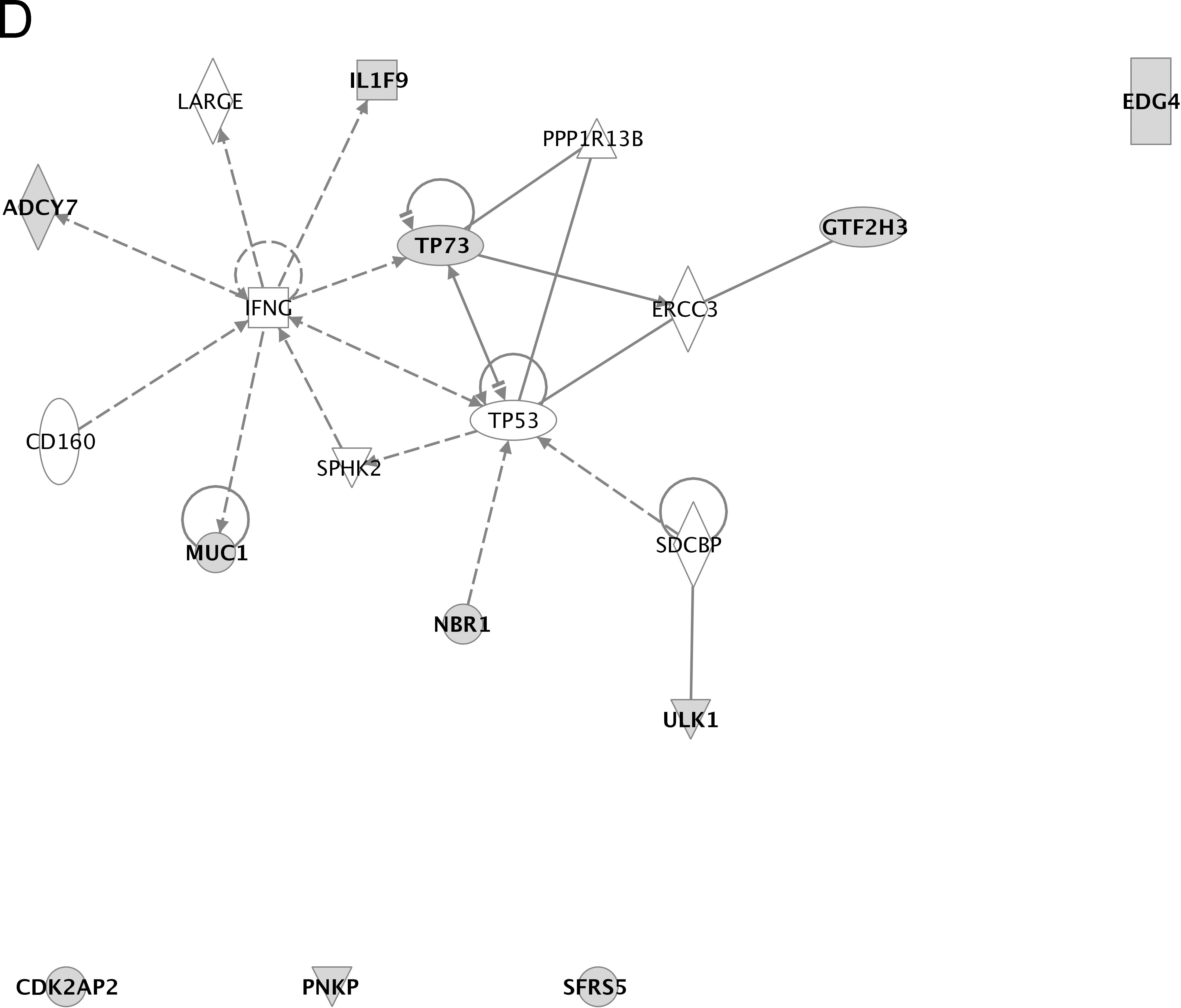

Supplement: Additional file 7 — Additional plausible network 5 in cluster 1. [file 1471-2105-8-S7-S2-S7.jpg]

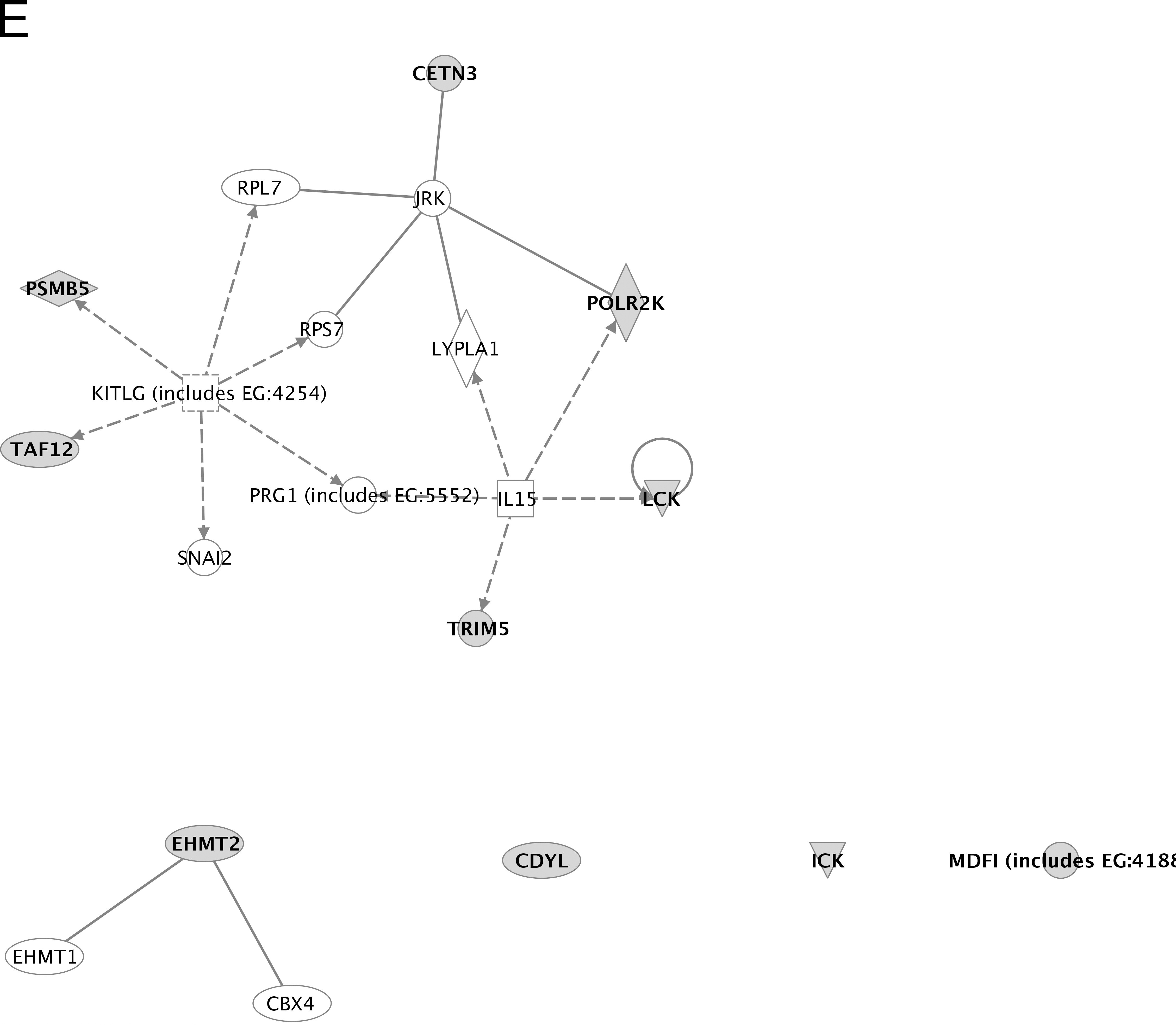

Supplement: Additional file 8 — Additional plausible network 6 in cluster 1. [file 1471-2105-8-S7-S2-S8.jpg]

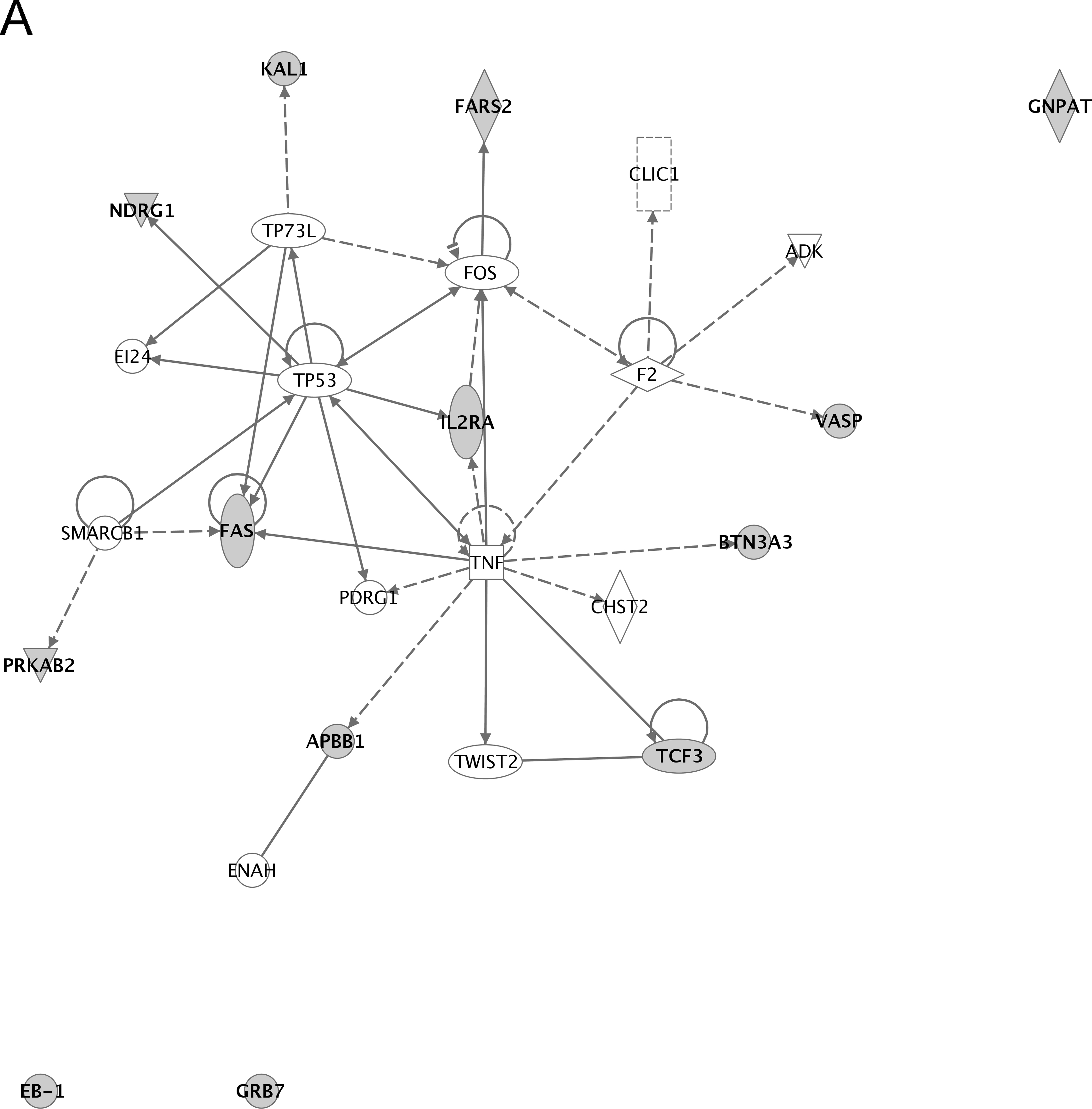

Supplement: Additional file 9 — Plausible network for Cluster 2, 1 hour post infection, up-regulated [file 1471-2105-8-S7-S2-S9.jpg]

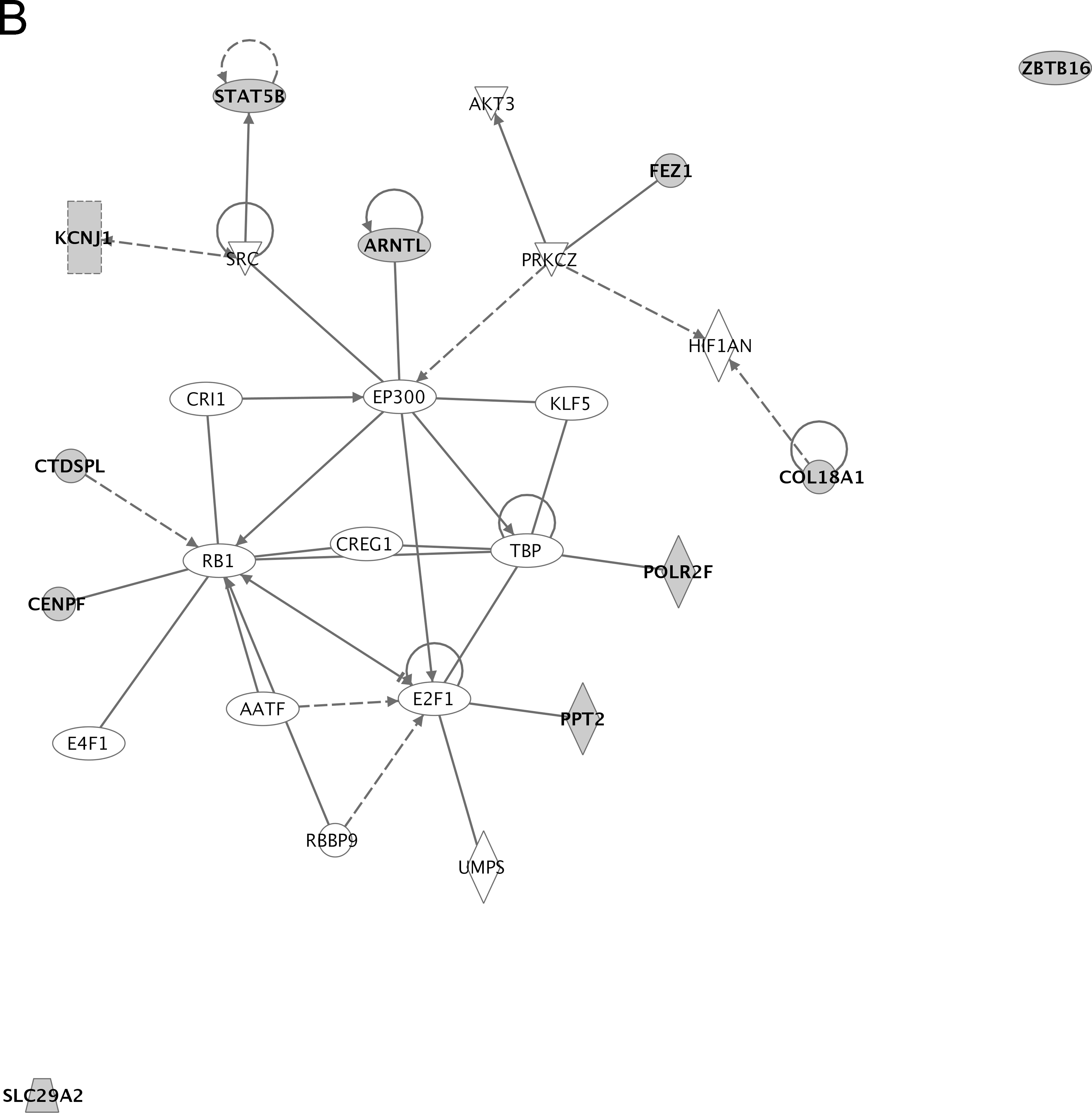

Supplement: Additional file 10 — Plausible network for Cluster 3, 1.5 hours post infection, up-regulated [file 1471-2105-8-S7-S2-S10.jpg]

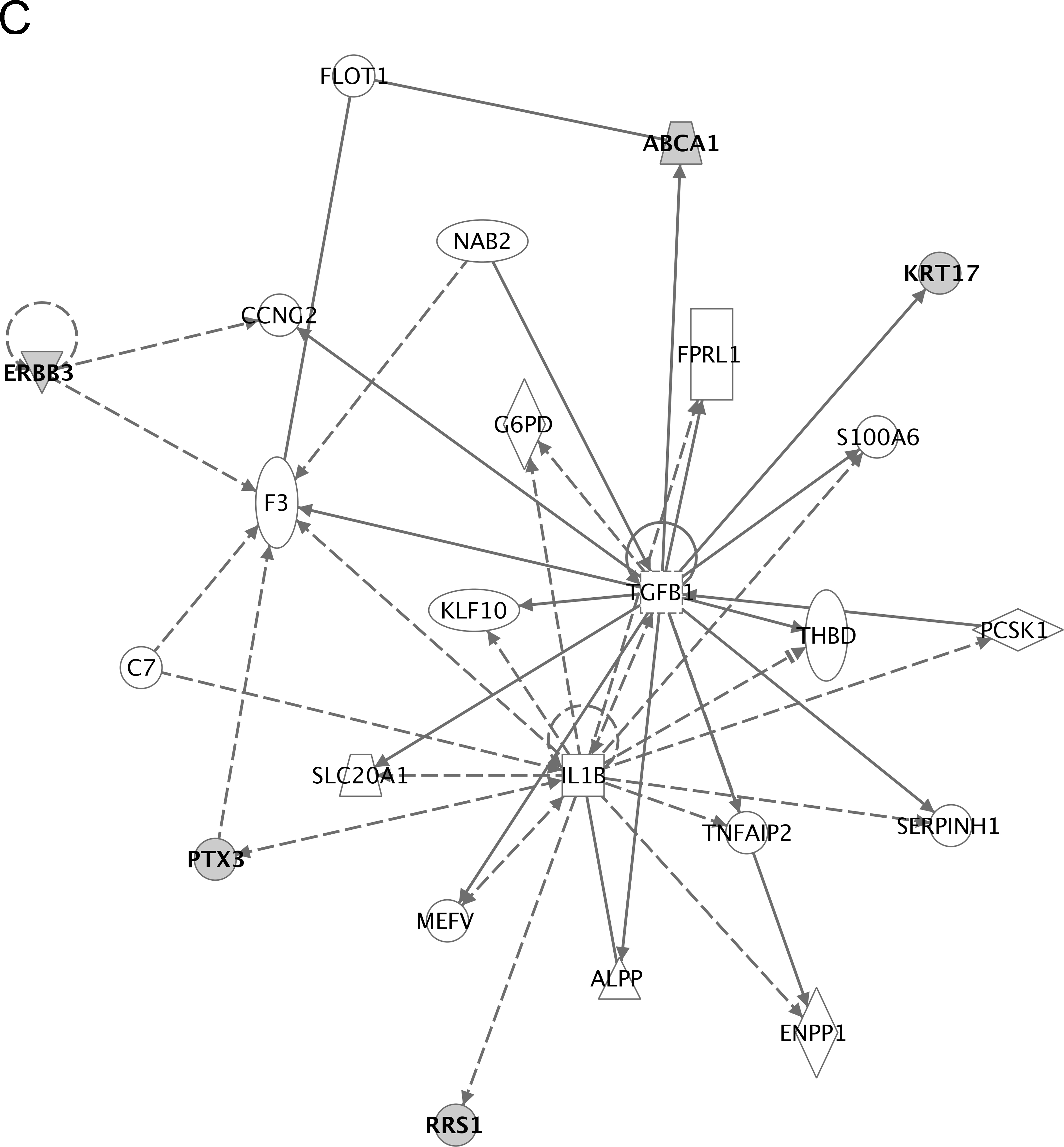

Supplement: Additional file 11 — Plausible network for Cluster 4, 1.5 hours post infection, down-regulated [file 1471-2105-8-S7-S2-S11.jpg]

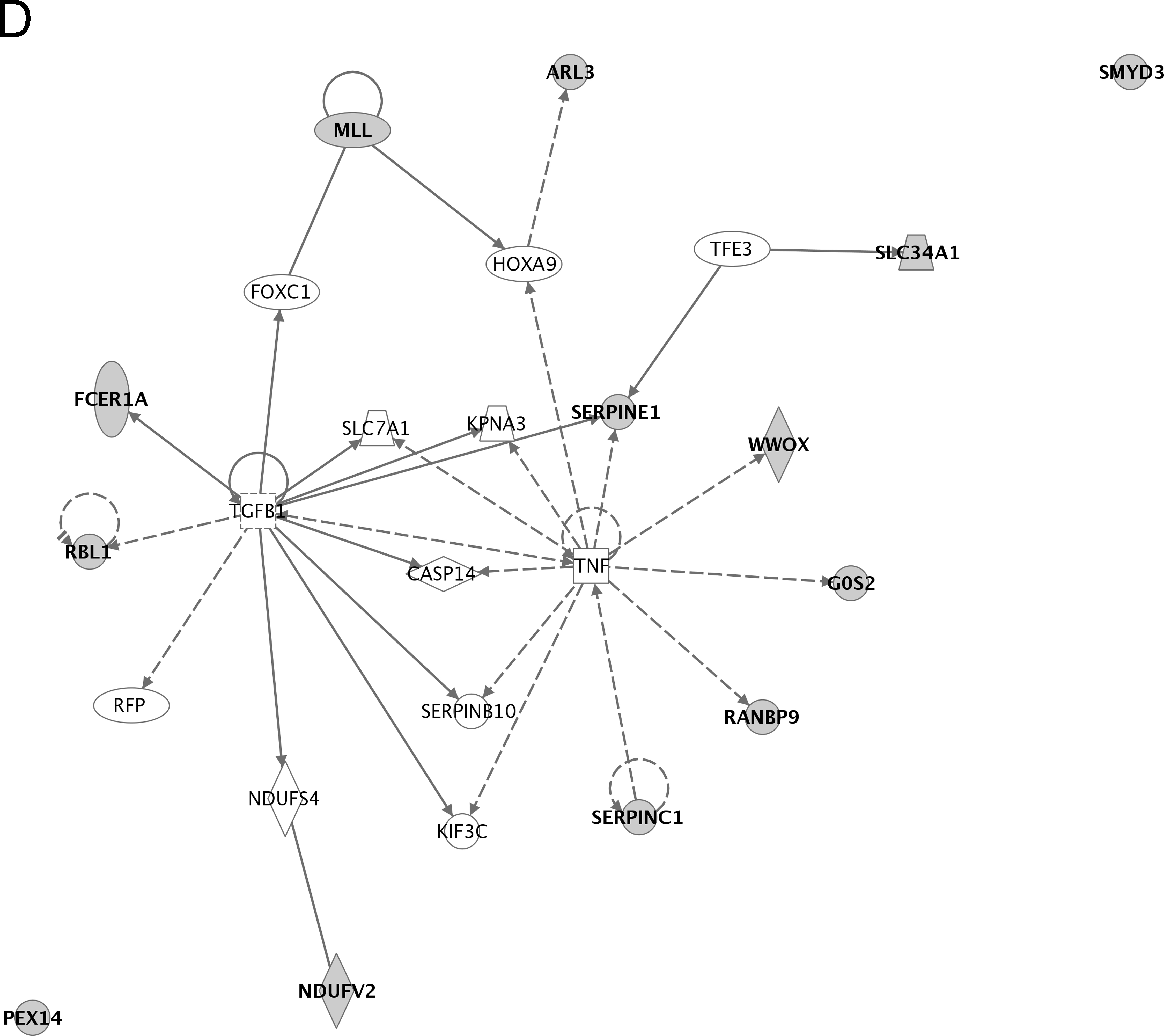

Supplement: Additional file 12 — Plausible network for Cluster 5, 4 hours post infection, up-regulated [file 1471-2105-8-S7-S2-S12.jpg]

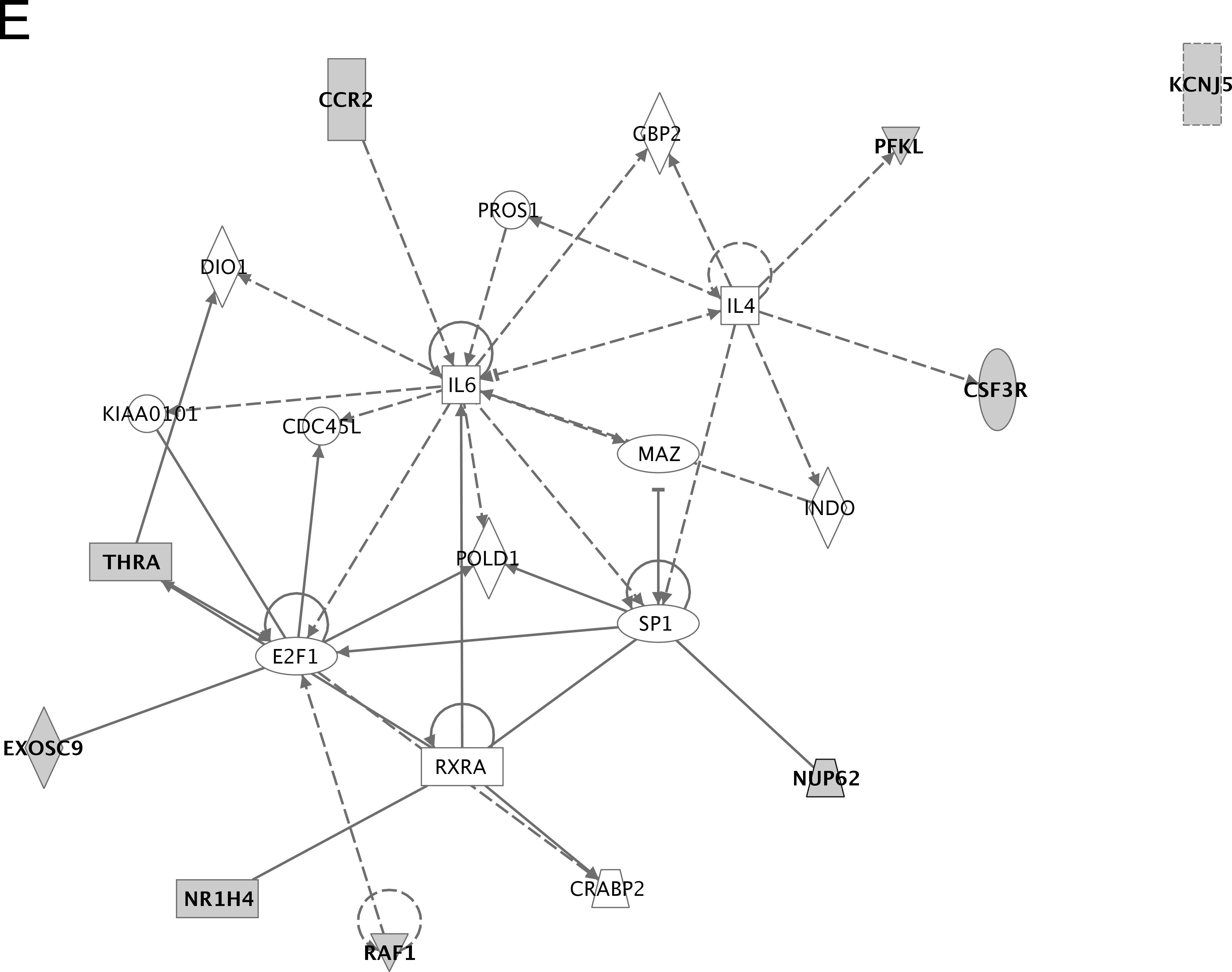

Supplement: Additional file 13 — Plausible network for Cluster 6, 4 hours post infection, down-regulated [file 1471-2105-8-S7-S2-S13.jpg]

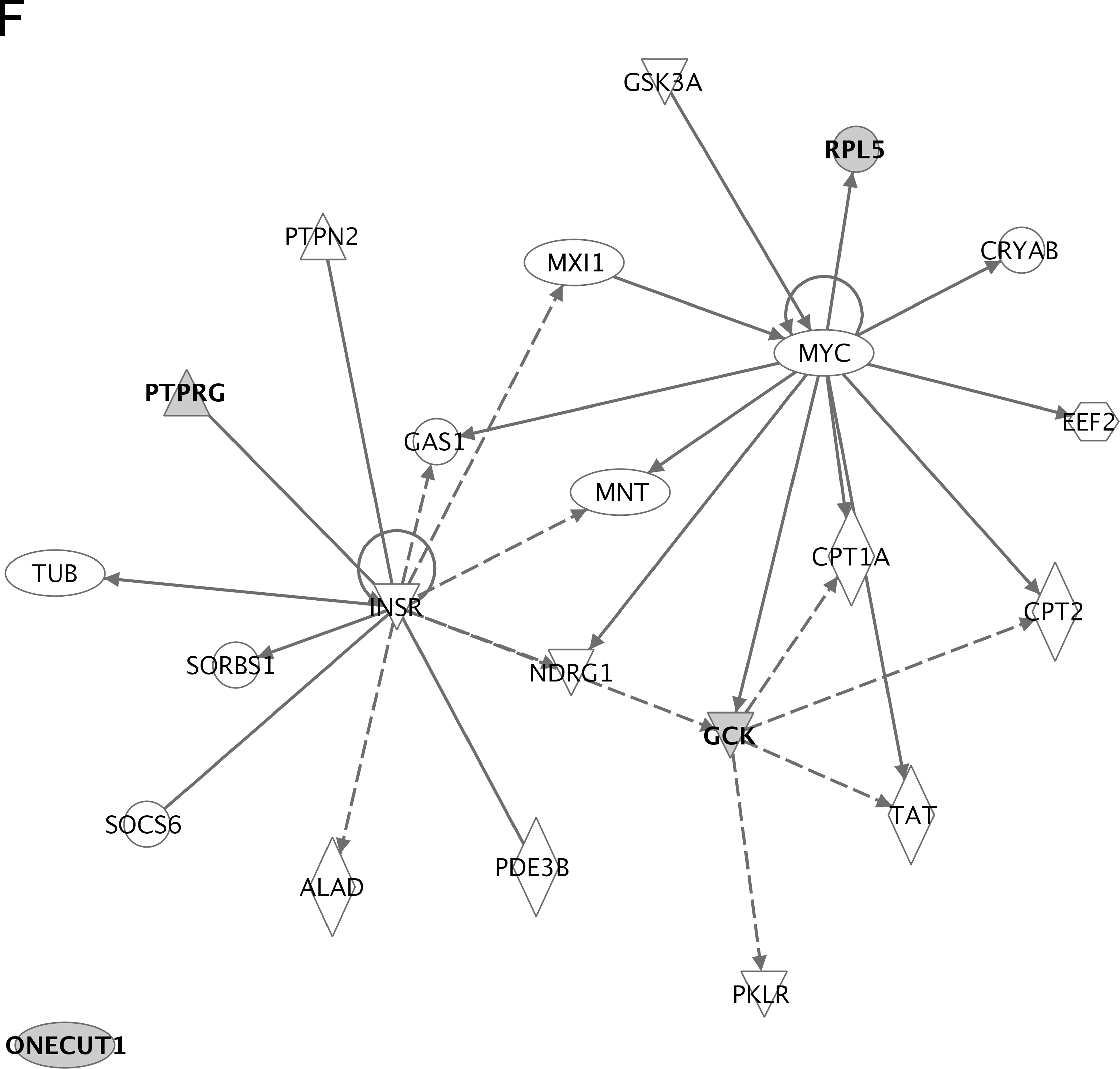

Supplement: Additional file 14 — Plausible network for Cluster 8, 8 hours post infection, up-regulated [file 1471-2105-8-S7-S2-S14.jpg]

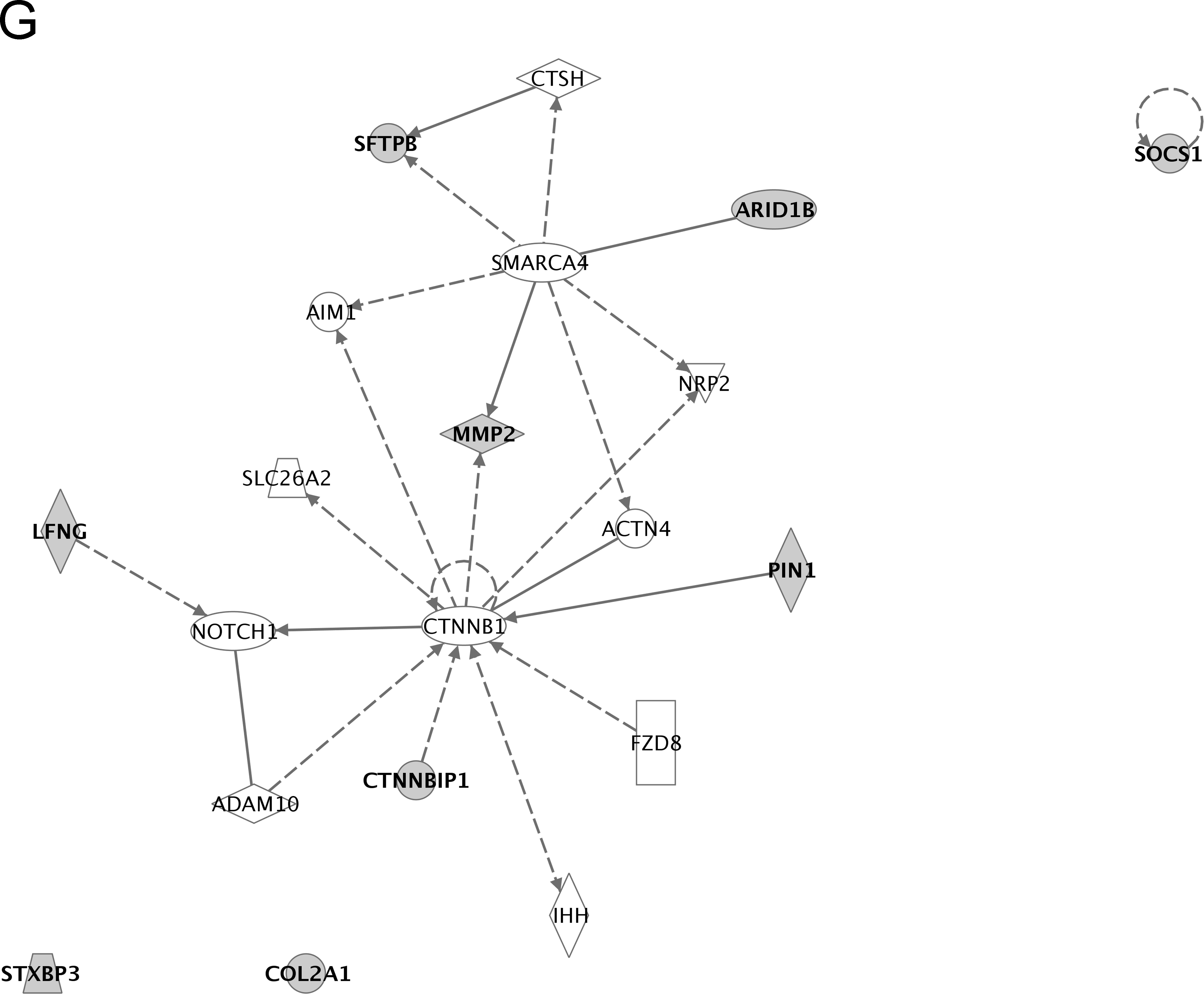

Supplement: Additional file 15 — Plausible network for Cluster 9, 8 hours post infection, down-regulated [file 1471-2105-8-S7-S2-S15.jpg]

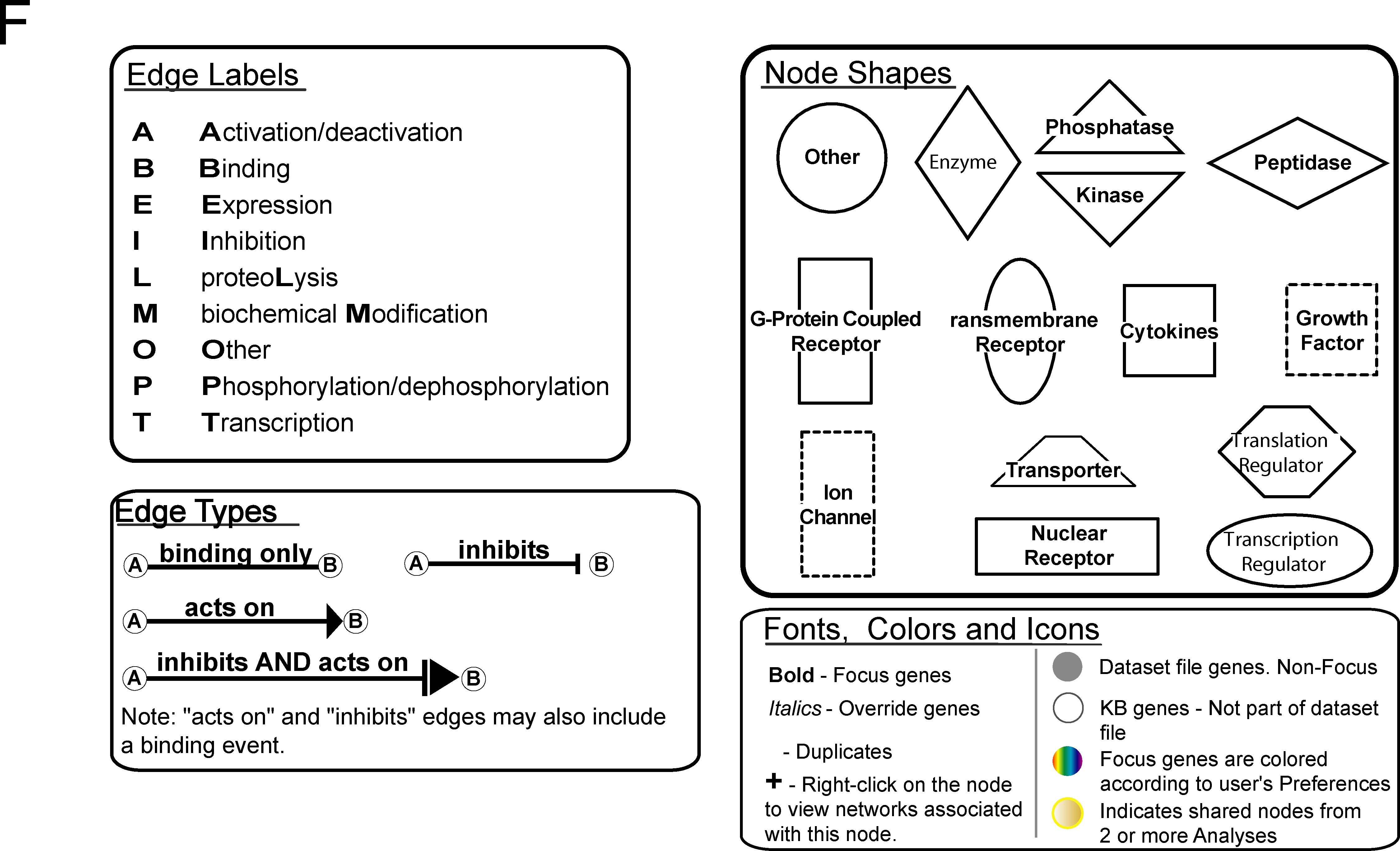

Supplement: Additional file 16 — Legends for IPA generated networks [file 1471-2105-8-S7-S2-S16.jpg]
