# Supplementary material for: Systems biology approach for mapping the response of human urothelial cells to infection by Enterococcus faecalis
Source: BMC Bioinformatics. 2007 Nov 1;8(Suppl 7):S2. doi: 10.1186/1471-2105-8-S7-S2 (PMC2099488; doi:10.1186/1471-2105-8-S7-S2)
Supplement: Additional file 2 — Full list of 239 VHV genes in different clusters. GenBank accession numbers, gene names and description provided. [file 1471-2105-8-S7-S2-S2.doc]

Table 1 suppl. Full list of 239 VHV genes in different clusters.

| **GB Acc** | **Gene Name** | **Description** | **Clusters** | **Time** |
| --- | --- | --- | --- | --- |
| *AK025051* | [*ACTR2*](http://www.genecards.org/cgi-bin/carddisp.pl?gene=ACTR2) | ARP2 actin-related protein 2 homolog (yeast) | 1 | 0-0.5h up |
| *AK001731* | [*C12orf23*](http://www.genecards.org/cgi-bin/carddisp.pl?gene=C12orf23) | Chromosome 12 open reading frame 23 | 1 | 0-0.5h up |
| *NM_016140* | [*CGI-38*](http://www.genecards.org/cgi-bin/carddisp.pl?gene=CGI-38) | Brain specific protein | 1 | 0-0.5h up |
| *AF368463* | [*CPM*](http://www.genecards.org/cgi-bin/carddisp.pl?gene=CPM) | Carboxypeptidase M | 1 | 0-0.5h up |
| *NM_003647* | [*DGKE*](http://www.genecards.org/cgi-bin/carddisp.pl?gene=DGKE) | Diacylglycerol kinase, epsilon 64kDa | 1 | 0-0.5h up |
| *AK054680* | [*FLJ22471*](http://www.genecards.org/cgi-bin/carddisp.pl?gene=FLJ22471) | Limkain beta 2 | 1 | 0-0.5h up |
| *AK023815* | [*GALNT10*](http://www.genecards.org/cgi-bin/carddisp.pl?gene=GALNT10) | Hypothetical protein FLJ38109 | 1 | 0-0.5h up |
| *NM_018649* | [*H2AFY2*](http://www.genecards.org/cgi-bin/carddisp.pl?gene=H2AFY2) | H2A histone family, member Y2 | 1 | 0-0.5h up |
| *AF098666* | [*LOC197350*](http://www.genecards.org/cgi-bin/carddisp.pl?gene=LOC197350) | Hypothetical protein LOC197350 | 1 | 0-0.5h up |
| *BC012855* | [*LRRC51*](http://www.genecards.org/cgi-bin/carddisp.pl?gene=LRRC51) | Leucine rich repeat containing 51 | 1 | 0-0.5h up |
| *NM_003550* | [*MAD1L1*](http://www.genecards.org/cgi-bin/carddisp.pl?gene=MAD1L1) | MAD1 mitotic arrest deficient-like 1 (yeast) | 1 | 0-0.5h up |
| *BC016950* | [*PHOSPHO2*](http://www.genecards.org/cgi-bin/carddisp.pl?gene=PHOSPHO2) | Kelch-like 23 (Drosophila) | 1 | 0-0.5h up |
| *NM_003557* | [*PIP5K1A*](http://www.genecards.org/cgi-bin/carddisp.pl?gene=PIP5K1A) | Phosphatidylinositol-4-phosphate 5-kinase, type I, alpha | 1 | 0-0.5h up |
| *NM_000942* | [*PPIB*](http://www.genecards.org/cgi-bin/carddisp.pl?gene=PPIB) | Peptidylprolyl isomerase B (cyclophilin B) | 1 | 0-0.5h up |
| *U36759* | [*PTCRA*](http://www.genecards.org/cgi-bin/carddisp.pl?gene=PTCRA) | Pre T-cell antigen receptor alpha | 1 | 0-0.5h up |
| *AK057320* | [*PTPRN2*](http://www.genecards.org/cgi-bin/carddisp.pl?gene=PTPRN2) | Protein tyrosine phosphatase, receptor type, N polypeptide 2 | 1 | 0-0.5h up |
| *NM_053285* | [*TEKT1*](http://www.genecards.org/cgi-bin/carddisp.pl?gene=TEKT1) | Tektin 1 | 1 | 0-0.5h up |
| *NM_032813* | [*TMTC4*](http://www.genecards.org/cgi-bin/carddisp.pl?gene=TMTC4) | Transmembrane and tetratricopeptide repeat containing 4 | 1 | 0-0.5h up |
| *AF017336* | *N/A* |  | 1 | 0-0.5h up |
| *NM_032585* | [*TTTY6*](http://www.genecards.org/cgi-bin/carddisp.pl?gene=TTTY6&search=NM_032585) | testis-specific transcript, Y-linked 6 | 1 | 0-0.5h up |
| *NM_001964* | [*EGR1*](http://www.genecards.org/cgi-bin/carddisp.pl?gene=EGR1) | Early growth response 1 | 2 | 1h up |
| *AK055613* | [*IKIP*](http://www.genecards.org/cgi-bin/carddisp.pl?gene=IKIP) | IKK interacting protein | 2 | 1h up |
| *D63480* | [*KIAA0146*](http://www.genecards.org/cgi-bin/carddisp.pl?gene=KIAA0146) | KIAA0146 protein | 2 | 1h up |
| *AK024302* | [*OCIAD1*](http://www.genecards.org/cgi-bin/carddisp.pl?gene=OCIAD1) | OCIA domain containing 1 | 2 | 1h up |
| *NM_003630* | [*PEX3*](http://www.genecards.org/cgi-bin/carddisp.pl?gene=PEX3) | Peroxisomal biogenesis factor 3 | 2 | 1h up |
| *NM_001198* | [*PRDM1*](http://www.genecards.org/cgi-bin/carddisp.pl?gene=PRDM1) | PR domain containing 1, with ZNF domain | 2 | 1h up |
| *NM_024638* | [*QTRTD1*](http://www.genecards.org/cgi-bin/carddisp.pl?gene=QTRTD1) | Queuine tRNA-ribosyltransferase domain containing 1 | 2 | 1h up |
| *NM_004850* | [*ROCK2*](http://www.genecards.org/cgi-bin/carddisp.pl?gene=ROCK2) | Rho-associated, coiled-coil containing protein kinase 2 | 2 | 1h up |
| *BC011566* | [*SRC*](http://www.genecards.org/cgi-bin/carddisp.pl?gene=SRC) | V-src sarcoma (Schmidt-Ruppin A-2) viral oncogene homolog (avian) | 2 | 1h up |
| *NM_033034* | [*TRIM5*](http://www.genecards.org/cgi-bin/carddisp.pl?gene=TRIM5) | Tripartite motif-containing pseudogene 1 | 2 | 1h up |
| *NM_058167* | [*UBE2J2*](http://www.genecards.org/cgi-bin/carddisp.pl?gene=UBE2J2) | Ubiquitin-conjugating enzyme E2, J2 (UBC6 homolog, yeast) | 2 | 1h up |
| *AB046809* | [*ZFYVE1*](http://www.genecards.org/cgi-bin/carddisp.pl?gene=ZFYVE1) | Zinc finger, FYVE domain containing 1 | 2 | 1h up |
| *AF145204* | [*ANKS1B*](http://www.genecards.org/cgi-bin/carddisp.pl?gene=ANKS1B) | Ankyrin repeat and sterile alpha motif domain containing 1B | 3 | 1.5h up |
| *NM_001164* | [*APBB1*](http://www.genecards.org/cgi-bin/carddisp.pl?gene=APBB1) | Amyloid beta (A4) precursor protein-binding, family B, member 1 (Fe65) | 3 | 1.5h up |
| *NM_006994* | [*BTN3A3*](http://www.genecards.org/cgi-bin/carddisp.pl?gene=BTN3A3) | Butyrophilin, subfamily 3, member A3 | 3 | 1.5h up |
| *AK057922* | [*CDH24*](http://www.genecards.org/cgi-bin/carddisp.pl?gene=CDH24) | Cadherin-like 24 | 3 | 1.5h up |
| *BI825261* | [*CST9L*](http://www.genecards.org/cgi-bin/carddisp.pl?gene=CST9L) | Cystatin 9-like (mouse) | 3 | 1.5h up |
| *AK001146* | [*FARS2*](http://www.genecards.org/cgi-bin/carddisp.pl?gene=FARS2) | Phenylalanine-tRNA synthetase 2 (mitochondrial) | 3 | 1.5h up |
| *NM_000043* | [*FAS*](http://www.genecards.org/cgi-bin/carddisp.pl?gene=FAS) | Fas (TNF receptor superfamily, member 6) | 3 | 1.5h up |
| *NM_024705* | [*FLJ13639*](http://www.genecards.org/cgi-bin/carddisp.pl?gene=FLJ13639) | Hypothetical protein FLJ13639 | 3 | 1.5h up |
| *AL512731* | [*FLJ34922*](http://www.genecards.org/cgi-bin/carddisp.pl?gene=FLJ34922) | Likely ortholog of mouse schlafen 8/9 | 3 | 1.5h up |
| *NM_014236* | [*GNPAT*](http://www.genecards.org/cgi-bin/carddisp.pl?gene=GNPAT) | Glyceronephosphate O-acyltransferase | 3 | 1.5h up |
| *AB007935* | [*IGSF3*](http://www.genecards.org/cgi-bin/carddisp.pl?gene=IGSF3) | Immunoglobulin superfamily, member 3 | 3 | 1.5h up |
| *NM_000417* | [*IL2RA*](http://www.genecards.org/cgi-bin/carddisp.pl?gene=IL2RA) | Interleukin 2 receptor, alpha | 3 | 1.5h up |
| *NM_000216* | [*KAL1*](http://www.genecards.org/cgi-bin/carddisp.pl?gene=KAL1) | Kallmann syndrome 1 sequence | 3 | 1.5h up |
| *BC016711* | [*LYPLAL1*](http://www.genecards.org/cgi-bin/carddisp.pl?gene=LYPLAL1) | Lysophospholipase-like 1 | 3 | 1.5h up |
| *AB011168* | [*MAPKBP1*](http://www.genecards.org/cgi-bin/carddisp.pl?gene=MAPKBP1) | Mitogen activated protein kinase binding protein 1 | 3 | 1.5h up |
| *AK000245* | [*MOGAT2*](http://www.genecards.org/cgi-bin/carddisp.pl?gene=MOGAT2) | Monoacylglycerol O-acyltransferase 2 | 3 | 1.5h up |
| *NM_006096* | [*NDRG1*](http://www.genecards.org/cgi-bin/carddisp.pl?gene=NDRG1) | N-myc downstream regulated gene 1 | 3 | 1.5h up |
| *NM_005399* | [*PRKAB2*](http://www.genecards.org/cgi-bin/carddisp.pl?gene=PRKAB2) | Protein kinase, AMP-activated, beta 2 non-catalytic subunit | 3 | 1.5h up |
| *AK054983* | [*RPH3AL*](http://www.genecards.org/cgi-bin/carddisp.pl?gene=RPH3AL) | Rabphilin 3A-like (without C2 domains) | 3 | 1.5h up |
| *AK024292* | [*SGOL1*](http://www.genecards.org/cgi-bin/carddisp.pl?gene=SGOL1) | Shugoshin-like 1 (S. pombe) | 3 | 1.5h up |
| *AB058735* | [*SPIRE2*](http://www.genecards.org/cgi-bin/carddisp.pl?gene=SPIRE2) | Spire homolog 2 (Drosophila) | 3 | 1.5h up |
| *AK055767* | [*STXBP2*](http://www.genecards.org/cgi-bin/carddisp.pl?gene=STXBP2) | Syntaxin binding protein 2 | 3 | 1.5h up |
| *M31523* | [*TCF3*](http://www.genecards.org/cgi-bin/carddisp.pl?gene=TCF3) | Transcription factor 3 (E2A immunoglobulin enhancer binding factors E12/E47) | 3 | 1.5h up |
| *NM_003449* | [*TRIM26*](http://www.genecards.org/cgi-bin/carddisp.pl?gene=TRIM26) | Tripartite motif-containing 26 | 3 | 1.5h up |
| *NM_003370* | [*VASP*](http://www.genecards.org/cgi-bin/carddisp.pl?gene=VASP) | Vasodilator-stimulated phosphoprotein | 3 | 1.5h up |
| *NM_025052* | [*YSK4*](http://www.genecards.org/cgi-bin/carddisp.pl?gene=YSK4) | Yeast Sps1/Ste20-related kinase 4 (S. cerevisiae) | 3 | 1.5h up |
| *AF130047* | *N/A* |  | 3 | 1.5h up |
| *AK054761* | *N/A* | CDNA FLJ30199 fis, clone BRACE2001453, weakly similar to 36.4 KDA PROLINE-RICH PROTEIN | 3 | 1.5h up |
| *AK057640* | [*GRB7*](http://www.genecards.org/cgi-bin/carddisp.pl?gene=GRB7&search=AK057640) | growth factor receptor-bound protein 7 | 3 | 1.5h up |
| *AL050039* | *N/A* | MRNA; cDNA DKFZp566K0324 (from clone DKFZp566K0324) | 3 | 1.5h up |
| *AL139174* | *N/A* |  | 3 | 1.5h up |
| *NM_001178* | [*ARNTL*](http://www.genecards.org/cgi-bin/carddisp.pl?gene=ARNTL) | Aryl hydrocarbon receptor nuclear translocator-like | 4 | 1.5h down |
| *AL133014* | [*C7orf20*](http://www.genecards.org/cgi-bin/carddisp.pl?gene=C7orf20) | Chromosome 7 open reading frame 20 | 4 | 1.5h down |
| *AL122120* | [*CCDC39*](http://www.genecards.org/cgi-bin/carddisp.pl?gene=CCDC39) | Coiled-coil domain containing 39 | 4 | 1.5h down |
| *AK055202* | [*CDH26*](http://www.genecards.org/cgi-bin/carddisp.pl?gene=CDH26) | Cadherin-like 26 | 4 | 1.5h down |
| *NM_016343* | [*CENPF*](http://www.genecards.org/cgi-bin/carddisp.pl?gene=CENPF) | Centromere protein F, 350/400ka (mitosin) | 4 | 1.5h down |
| *AF113017* | [*CHD9*](http://www.genecards.org/cgi-bin/carddisp.pl?gene=CHD9) | Chromodomain helicase DNA binding protein 9 | 4 | 1.5h down |
| *AF018081* | [*COL18A1*](http://www.genecards.org/cgi-bin/carddisp.pl?gene=COL18A1) | Collagen, type XVIII, alpha 1 | 4 | 1.5h down |
| *NM_005808* | [*CTDSPL*](http://www.genecards.org/cgi-bin/carddisp.pl?gene=CTDSPL) | CTD (carboxy-terminal domain, RNA polymerase II, polypeptide A) small phosphatase-like | 4 | 1.5h down |
| *NM_022549* | [*FEZ1*](http://www.genecards.org/cgi-bin/carddisp.pl?gene=FEZ1) | Fasciculation and elongation protein zeta 1 (zygin I) | 4 | 1.5h down |
| *AK055708* | [*FLJ22313*](http://www.genecards.org/cgi-bin/carddisp.pl?gene=FLJ22313) | Hypothetical protein FLJ22313 | 4 | 1.5h down |
| *AK056513* | [*FLJ31951*](http://www.genecards.org/cgi-bin/carddisp.pl?gene=FLJ31951) | Hypothetical protein FLJ31951 | 4 | 1.5h down |
| *NM_000220* | [*KCNJ1*](http://www.genecards.org/cgi-bin/carddisp.pl?gene=KCNJ1) | Potassium inwardly-rectifying channel, subfamily J, member 1 | 4 | 1.5h down |
| *AB014574* | [*KIAA0674*](http://www.genecards.org/cgi-bin/carddisp.pl?gene=KIAA0674) | KIAA0674 | 4 | 1.5h down |
| *AK055370* | [*LOC283658*](http://www.genecards.org/cgi-bin/carddisp.pl?gene=LOC283658) | Hypothetical protein LOC283658 | 4 | 1.5h down |
| *AK056895* | [*LOC400924*](http://www.genecards.org/cgi-bin/carddisp.pl?gene=LOC400924) | Hypothetical gene supported by AK056895 | 4 | 1.5h down |
| *NM_030578* | [*MGC4093*](http://www.genecards.org/cgi-bin/carddisp.pl?gene=MGC4093) | Hypothetical protein MGC4093 | 4 | 1.5h down |
| *NM_004289* | [*NFE2L3*](http://www.genecards.org/cgi-bin/carddisp.pl?gene=NFE2L3) | Nuclear factor (erythroid-derived 2)-like 3 | 4 | 1.5h down |
| *NM_013248* | [*NXT1*](http://www.genecards.org/cgi-bin/carddisp.pl?gene=NXT1) | NTF2-like export factor 1 | 4 | 1.5h down |
| *NM_021974* | [*POLR2F*](http://www.genecards.org/cgi-bin/carddisp.pl?gene=POLR2F) | Polymerase (RNA) II (DNA directed) polypeptide F | 4 | 1.5h down |
| *BC016984* | [*PPIL4*](http://www.genecards.org/cgi-bin/carddisp.pl?gene=PPIL4) | Peptidylprolyl isomerase (cyclophilin)-like 4 | 4 | 1.5h down |
| *AF020544* | [*PPT2*](http://www.genecards.org/cgi-bin/carddisp.pl?gene=PPT2) | Palmitoyl-protein thioesterase 2 | 4 | 1.5h down |
| *AK001508* | [*PYCR2*](http://www.genecards.org/cgi-bin/carddisp.pl?gene=PYCR2) | Pyrroline-5-carboxylate reductase family, member 2 | 4 | 1.5h down |
| *BC006132* | [*PYGO2*](http://www.genecards.org/cgi-bin/carddisp.pl?gene=PYGO2) | Pygopus homolog 2 (Drosophila) | 4 | 1.5h down |
| *AK024868* | [*RAB6IP2*](http://www.genecards.org/cgi-bin/carddisp.pl?gene=RAB6IP2) | RAB6 interacting protein 2 | 4 | 1.5h down |
| *NM_001532* | [*SLC29A2*](http://www.genecards.org/cgi-bin/carddisp.pl?gene=SLC29A2) | Solute carrier family 29 (nucleoside transporters), member 2 | 4 | 1.5h down |
| *NM_015948* | [*SLC35B3*](http://www.genecards.org/cgi-bin/carddisp.pl?gene=SLC35B3) | Solute carrier family 35, member B3 | 4 | 1.5h down |
| *NM_003116* | [*SPAG4*](http://www.genecards.org/cgi-bin/carddisp.pl?gene=SPAG4) | Sperm associated antigen 4 | 4 | 1.5h down |
| *AL080218* | [*STAT5B*](http://www.genecards.org/cgi-bin/carddisp.pl?gene=STAT5B) | Signal transducer and activator of transcription 5B | 4 | 1.5h down |
| *NM_014760* | [*TATDN2*](http://www.genecards.org/cgi-bin/carddisp.pl?gene=TATDN2) | TatD DNase domain containing 2 | 4 | 1.5h down |
| *AK057366* | [*TRIM50A*](http://www.genecards.org/cgi-bin/carddisp.pl?gene=TRIM50A) | Tripartite motif-containing 50A | 4 | 1.5h down |
| *AK024303* | [*UCA1*](http://www.genecards.org/cgi-bin/carddisp.pl?gene=UCA1) | UCA1 protein | 4 | 1.5h down |
| *AK023845* | [*USP34*](http://www.genecards.org/cgi-bin/carddisp.pl?gene=USP34) | Ubiquitin specific peptidase 34 | 4 | 1.5h down |
| *NM_014709* | [*USP34*](http://www.genecards.org/cgi-bin/carddisp.pl?gene=USP34) | Ubiquitin specific peptidase 34 | 4 | 1.5h down |
| *NM_006006* | [*ZBTB16*](http://www.genecards.org/cgi-bin/carddisp.pl?gene=ZBTB16) | Zinc finger and BTB domain containing 16 | 4 | 1.5h down |
| *AF279773* | *N/A* | Clone N1 NTera2D1 teratocarcinoma mRNA | 4 | 1.5h down |
| *AK022254* | [*ABCA1*](http://www.genecards.org/cgi-bin/carddisp.pl?gene=ABCA1) | ATP-binding cassette, sub-family A (ABC1), member 1 | 5 | 4h up |
| *NM_014256* | [*B3GNT3*](http://www.genecards.org/cgi-bin/carddisp.pl?gene=B3GNT3) | UDP-GlcNAc:betaGal beta-1,3-N-acetylglucosaminyltransferase 3 | 5 | 4h up |
| *NM_001900* | [*CST5*](http://www.genecards.org/cgi-bin/carddisp.pl?gene=CST5) | Cystatin D | 5 | 4h up |
| *U88358* | [*ERBB3*](http://www.genecards.org/cgi-bin/carddisp.pl?gene=ERBB3) | V-erb-b2 erythroblastic leukemia viral oncogene homolog 3 (avian) | 5 | 4h up |
| *AK027561* | [*FLJ10006*](http://www.genecards.org/cgi-bin/carddisp.pl?gene=FLJ10006) | Hypothetical protein LOC55677 | 5 | 4h up |
| *NM_001460* | [*FMO2*](http://www.genecards.org/cgi-bin/carddisp.pl?gene=FMO2) | Flavin containing monooxygenase 2 | 5 | 4h up |
| *NM_013345* | [*GPR132*](http://www.genecards.org/cgi-bin/carddisp.pl?gene=GPR132) | G protein-coupled receptor 132 | 5 | 4h up |
| *NM_007031* | [*HSF2BP*](http://www.genecards.org/cgi-bin/carddisp.pl?gene=HSF2BP) | Heat shock transcription factor 2 binding protein | 5 | 4h up |
| *NM_000422* | [*KRT17*](http://www.genecards.org/cgi-bin/carddisp.pl?gene=KRT17) | Keratin 17 | 5 | 4h up |
| *BC012173* | [*KTI12*](http://www.genecards.org/cgi-bin/carddisp.pl?gene=KTI12) | KTI12 homolog, chromatin associated (S. cerevisiae) | 5 | 4h up |
| *NM_032476* | [*MRPS6*](http://www.genecards.org/cgi-bin/carddisp.pl?gene=MRPS6) | Mitochondrial ribosomal protein S6 | 5 | 4h up |
| *NM_002852* | [*PTX3*](http://www.genecards.org/cgi-bin/carddisp.pl?gene=PTX3) | Pentraxin-related gene, rapidly induced by IL-1 beta | 5 | 4h up |
| *NM_002854* | [*PVALB*](http://www.genecards.org/cgi-bin/carddisp.pl?gene=PVALB) | Parvalbumin | 5 | 4h up |
| *NM_015169* | [*RRS1*](http://www.genecards.org/cgi-bin/carddisp.pl?gene=RRS1) | RRS1 ribosome biogenesis regulator homolog (S. cerevisiae) | 5 | 4h up |
| *AK055230* | [*TMEM74*](http://www.genecards.org/cgi-bin/carddisp.pl?gene=TMEM74) | Transmembrane protein 74 | 5 | 4h up |
| *BC010012* | [*WBP1*](http://www.genecards.org/cgi-bin/carddisp.pl?gene=WBP1) | WW domain binding protein 1 | 5 | 4h up |
| *BC014989* |  |  | 5 | 4h up |
| *NM_018985* | [*HCG4*](http://www.genecards.org/cgi-bin/carddisp.pl?gene=HCG4&search=NM_018985) | HLA complex group 4 | 5 | 4h up |
| *NM_001605* | [*AARS*](http://www.genecards.org/cgi-bin/carddisp.pl?gene=AARS) | Alanyl-tRNA synthetase | 6 | 4h down |
| *NM_001659* | [*ARF3*](http://www.genecards.org/cgi-bin/carddisp.pl?gene=ARF3) | ADP-ribosylation factor 3 | 6 | 4h down |
| *NM_004311* | [*ARL3*](http://www.genecards.org/cgi-bin/carddisp.pl?gene=ARL3) | ADP-ribosylation factor-like 3 | 6 | 4h down |
| *AF274940* | [*C12orf48*](http://www.genecards.org/cgi-bin/carddisp.pl?gene=C12orf48) | Chromosome 12 open reading frame 48 | 6 | 4h down |
| *AK026743* | [*C21orf96*](http://www.genecards.org/cgi-bin/carddisp.pl?gene=C21orf96) | Chromosome 21 open reading frame 96 | 6 | 4h down |
| *NM_003159* | [*CDKL5*](http://www.genecards.org/cgi-bin/carddisp.pl?gene=CDKL5) | Cyclin-dependent kinase-like 5 | 6 | 4h down |
| *NM_004395* | [*DBN1*](http://www.genecards.org/cgi-bin/carddisp.pl?gene=DBN1) | Drebrin 1 | 6 | 4h down |
| *NM_003859* | [*DPM1*](http://www.genecards.org/cgi-bin/carddisp.pl?gene=DPM1) | Dolichyl-phosphate mannosyltransferase polypeptide 1, catalytic subunit | 6 | 4h down |
| *AB019568* | [*EEF1A1*](http://www.genecards.org/cgi-bin/carddisp.pl?gene=EEF1A1) | eukaryotic translation elongation factor 1 alpha 1 | 6 | 4h down |
| *BC015185* | [*FCER1A*](http://www.genecards.org/cgi-bin/carddisp.pl?gene=FCER1A) | Fc fragment of IgE, high affinity I, receptor for; alpha polypeptide | 6 | 4h down |
| *BC011872* | [*FKBP9L*](http://www.genecards.org/cgi-bin/carddisp.pl?gene=FKBP9L) | FK506 binding protein 9-like | 6 | 4h down |
| *NM_015714* | [*G0S2*](http://www.genecards.org/cgi-bin/carddisp.pl?gene=G0S2) | G0/G1switch 2 | 6 | 4h down |
| *NM_020155* | [*GPR137*](http://www.genecards.org/cgi-bin/carddisp.pl?gene=GPR137) | G protein-coupled receptor 137 | 6 | 4h down |
| *NM_000637* | [*GSR*](http://www.genecards.org/cgi-bin/carddisp.pl?gene=GSR) | Glutathione reductase | 6 | 4h down |
| *D87077* | [*KIAA0240*](http://www.genecards.org/cgi-bin/carddisp.pl?gene=KIAA0240) | KIAA0240 | 6 | 4h down |
| *AB018319* | [*KIAA0776*](http://www.genecards.org/cgi-bin/carddisp.pl?gene=KIAA0776) | KIAA0776 | 6 | 4h down |
| *AL512697* | [*LOC400547*](http://www.genecards.org/cgi-bin/carddisp.pl?gene=LOC400547) | Similar to C10orf94 protein | 6 | 4h down |
| *BC009233* | [*LOC92659*](http://www.genecards.org/cgi-bin/carddisp.pl?gene=LOC92659) | Hypothetical protein BC009233 | 6 | 4h down |
| *NM_002408* | [*MGAT2*](http://www.genecards.org/cgi-bin/carddisp.pl?gene=MGAT2) | Mannosyl (alpha-1,6-)-glycoprotein beta-1,2-N-acetylglucosaminyltransferase | 6 | 4h down |
| *AK058000* | [*MGC5509*](http://www.genecards.org/cgi-bin/carddisp.pl?gene=MGC5509) | Hypothetical protein MGC5509 | 6 | 4h down |
| *L04731* | [*MLL*](http://www.genecards.org/cgi-bin/carddisp.pl?gene=MLL) | Myeloid/lymphoid or mixed-lineage leukemia (trithorax homolog, Drosophila) | 6 | 4h down |
| *NM_021074* | [*NDUFV2*](http://www.genecards.org/cgi-bin/carddisp.pl?gene=NDUFV2) | NADH dehydrogenase (ubiquinone) flavoprotein 2, 24kDa | 6 | 4h down |
| *NM_004565* | [*PEX14*](http://www.genecards.org/cgi-bin/carddisp.pl?gene=PEX14) | Peroxisomal biogenesis factor 14 | 6 | 4h down |
| *NM_005493* | [*RANBP9*](http://www.genecards.org/cgi-bin/carddisp.pl?gene=RANBP9) | RAN binding protein 9 | 6 | 4h down |
| *NM_002895* | [*RBL1*](http://www.genecards.org/cgi-bin/carddisp.pl?gene=RBL1) | Retinoblastoma-like 1 (p107) | 6 | 4h down |
| *AJ243672* | [*S100A7L1*](http://www.genecards.org/cgi-bin/carddisp.pl?gene=S100A7L1) | S100 calcium binding protein A7-like 1 | 6 | 4h down |
| *NM_000488* | [*SERPINC1*](http://www.genecards.org/cgi-bin/carddisp.pl?gene=SERPINC1) | Serpin peptidase inhibitor, clade C (antithrombin), member 1 | 6 | 4h down |
| *NM_000602* | [*SERPINE1*](http://www.genecards.org/cgi-bin/carddisp.pl?gene=SERPINE1) | Serpin peptidase inhibitor, clade E (nexin, plasminogen activator inhibitor type 1), member 1 | 6 | 4h down |
| *AB051437* | [*SHANK3*](http://www.genecards.org/cgi-bin/carddisp.pl?gene=SHANK3) | SH3 and multiple ankyrin repeat domains 3 | 6 | 4h down |
| *NM_003052* | [*SLC34A1*](http://www.genecards.org/cgi-bin/carddisp.pl?gene=SLC34A1) | Solute carrier family 34 (sodium phosphate), member 1 | 6 | 4h down |
| *AF104032* | [*SLC7A5*](http://www.genecards.org/cgi-bin/carddisp.pl?gene=SLC7A5) | Solute carrier family 7 (cationic amino acid transporter, y+ system), member 5 | 6 | 4h down |
| *NM_022743* | [*SMYD3*](http://www.genecards.org/cgi-bin/carddisp.pl?gene=SMYD3) | SET and MYND domain containing 3 | 6 | 4h down |
| *AK026820* | [*ST3GAL1*](http://www.genecards.org/cgi-bin/carddisp.pl?gene=ST3GAL1) | ST3 beta-galactoside alpha-2,3-sialyltransferase 1 | 6 | 4h down |
| *NM_003165* | [*STXBP1*](http://www.genecards.org/cgi-bin/carddisp.pl?gene=STXBP1) | Syntaxin binding protein 1 | 6 | 4h down |
| *NM_003276* | [*TMPO*](http://www.genecards.org/cgi-bin/carddisp.pl?gene=TMPO) | Thymopoietin | 6 | 4h down |
| *AK024586* | [*TNC*](http://www.genecards.org/cgi-bin/carddisp.pl?gene=TNC) | Tenascin C (hexabrachion) | 6 | 4h down |
| *AL050145* | [*WWOX*](http://www.genecards.org/cgi-bin/carddisp.pl?gene=WWOX) | B-box and SPRY domain containing | 6 | 4h down |
| *AK022842* | [*ZDHHC23*](http://www.genecards.org/cgi-bin/carddisp.pl?gene=ZDHHC23) | Zinc finger, DHHC-type containing 23 | 6 | 4h down |
| *AL110236* | [*ZF*](http://www.genecards.org/cgi-bin/carddisp.pl?gene=ZF) | HCF-binding transcription factor Zhangfei | 6 | 4h down |
| *AF212224* | [*CLK1*](http://www.genecards.org/cgi-bin/carddisp.pl?gene=CLK1&search=AF212224)  [*CLK4*](http://www.genecards.org/cgi-bin/carddisp.pl?gene=CLK4&search=AF212224) | CDC-like kinase 1, 4 | 6 | 4h down |
| *AK024111* | [*HCG11*](http://www.genecards.org/cgi-bin/carddisp.pl?gene=HCG11&search=AK024111) | HLA complex group 11 | 6 | 4h down |
| *AK056890* | *N/A* | CDNA FLJ32328 fis, clone PROST2004481 | 6 | 4h down |
| *NM_003832* | [*PSPHL*](http://www.genecards.org/cgi-bin/carddisp.pl?gene=PSPHL&search=NM_003832) | phosphoserine phosphatase-like | 6 | 4h down |
| *NM_005757* | [*MBNL2*](http://www.genecards.org/cgi-bin/carddisp.pl?gene=MBNL2&search=NM_005757) | muscleblind-like 2 (Drosophila) | 6 | 4h down |
| *NM_018615* | *N/A* |  | 6 | 4h down |
| *Y13187* | *N/A* |  | 6 | 4h down |
| *AK024871* | [*APBB2*](http://www.genecards.org/cgi-bin/carddisp.pl?gene=APBB2) | Amyloid beta (A4) precursor protein-binding, family B, member 2 (Fe65-like) | 7 | 6h up |
| *NM_031371* | [*ARID4B*](http://www.genecards.org/cgi-bin/carddisp.pl?gene=ARID4B) | AT rich interactive domain 4B (RBP1- like) | 7 | 6h up |
| *NM_001204* | [*BMPR2*](http://www.genecards.org/cgi-bin/carddisp.pl?gene=BMPR2) | Bone morphogenetic protein receptor, type II (serine/threonine kinase) | 7 | 6h up |
| *NM_032830* | [*CIRH1A*](http://www.genecards.org/cgi-bin/carddisp.pl?gene=CIRH1A) | Cirrhosis, autosomal recessive 1A (cirhin) | 7 | 6h up |
| *AF056085* | *GABBR2*  *GRP51* | Gamma-aminobutyric acid (GABA) B receptor, 2 | 7 | 6h up |
| *NM_004127* | [*GPS1*](http://www.genecards.org/cgi-bin/carddisp.pl?gene=GPS1) | G protein pathway suppressor 1 | 7 | 6h up |
| *NM_033626* | [*JM11*](http://www.genecards.org/cgi-bin/carddisp.pl?gene=JM11) | JM11 protein | 7 | 6h up |
| *AY043362* | [*KIF1B*](http://www.genecards.org/cgi-bin/carddisp.pl?gene=KIF1B) | Kinesin family member 1B | 7 | 6h up |
| *AF397397* | [*OLFM3*](http://www.genecards.org/cgi-bin/carddisp.pl?gene=OLFM3) | Olfactomedin 3 | 7 | 6h up |
| *BC015824* | [*P29*](http://www.genecards.org/cgi-bin/carddisp.pl?gene=P29) | SYF2 homolog, RNA splicing factor (S. cerevisiae) | 7 | 6h up |
| *AB037794* | [*STAMBPL1*](http://www.genecards.org/cgi-bin/carddisp.pl?gene=STAMBPL1) | STAM binding protein-like 1 | 7 | 6h up |
| *NM_000361* | [*THBD*](http://www.genecards.org/cgi-bin/carddisp.pl?gene=THBD) | Thrombomodulin | 7 | 6h up |
| *AK021722* |  |  | 7 | 6h up |
| *AY040326* | [*ANTXR2*](http://www.genecards.org/cgi-bin/carddisp.pl?gene=ANTXR2) | Anthrax toxin receptor 2 | 8 | 8h up |
| *NM_001644* | [*APOBEC1*](http://www.genecards.org/cgi-bin/carddisp.pl?gene=APOBEC1) | Apolipoprotein B mRNA editing enzyme, catalytic polypeptide 1 | 8 | 8h up |
| *AK001155* | [*ATP6V1D*](http://www.genecards.org/cgi-bin/carddisp.pl?gene=ATP6V1D) | ATPase, H+ transporting, lysosomal 34kDa, V1 subunit D | 8 | 8h up |
| *NM_000647* | [*CCR2*](http://www.genecards.org/cgi-bin/carddisp.pl?gene=CCR2) | Chemokine (C-C motif) receptor 2 | 8 | 8h up |
| *NM_000760* | [*CSF3R*](http://www.genecards.org/cgi-bin/carddisp.pl?gene=CSF3R) | Colony stimulating factor 3 receptor (granulocyte) | 8 | 8h up |
| *AF007147* | [*CUGBP2*](http://www.genecards.org/cgi-bin/carddisp.pl?gene=CUGBP2) | CUG triplet repeat, RNA binding protein 2 | 8 | 8h up |
| *J00146* | [*DHFRP1*](http://www.genecards.org/cgi-bin/carddisp.pl?gene=DHFRP1) | Dihydrofolate reductase pseudogene 1 | 8 | 8h up |
| *AB051510* | [*DLC1*](http://www.genecards.org/cgi-bin/carddisp.pl?gene=DLC1) | Deleted in liver cancer 1 | 8 | 8h up |
| *NM_005033* | [*EXOSC9*](http://www.genecards.org/cgi-bin/carddisp.pl?gene=EXOSC9) | Exosome component 9 | 8 | 8h up |
| *NM_000890* | [*KCNJ5*](http://www.genecards.org/cgi-bin/carddisp.pl?gene=KCNJ5) | Potassium inwardly-rectifying channel, subfamily J, member 5 | 8 | 8h up |
| *NM_005123* | [*NR1H4*](http://www.genecards.org/cgi-bin/carddisp.pl?gene=NR1H4) | Nuclear receptor subfamily 1, group H, member 4 | 8 | 8h up |
| *NM_016553* | [*NUP62*](http://www.genecards.org/cgi-bin/carddisp.pl?gene=NUP62) | Interleukin 4 induced 1 | 8 | 8h up |
| *NM_002626* | [*PFKL*](http://www.genecards.org/cgi-bin/carddisp.pl?gene=PFKL) | Phosphofructokinase, liver | 8 | 8h up |
| *AK055589* | [*RAF1*](http://www.genecards.org/cgi-bin/carddisp.pl?gene=RAF1) | V-raf-1 murine leukemia viral oncogene homolog 1 | 8 | 8h up |
| *NM_004768* | [*SFRS11*](http://www.genecards.org/cgi-bin/carddisp.pl?gene=SFRS11) | Splicing factor, arginine/serine-rich 11 | 8 | 8h up |
| *NM_003250* | [*THRA*](http://www.genecards.org/cgi-bin/carddisp.pl?gene=THRA) | Thyroid hormone receptor, alpha (erythroblastic leukemia viral (v-erb-a) oncogene homolog, avian) | 8 | 8h up |
| *NM_003692* | [*TMEFF1*](http://www.genecards.org/cgi-bin/carddisp.pl?gene=TMEFF1) | Transmembrane protein with EGF-like and two follistatin-like domains 1 | 8 | 8h up |
| *BC009366* | *N/A* | CDNA clone IMAGE:4110850 | 8 | 8h up |
| *NM_032240* | [*FLJ23519*](http://www.genecards.org/cgi-bin/carddisp.pl?gene=FLJ23519&search=NM_032240) | hypothetical protein FLJ23519 | 8 | 8h up |
| *NM_001097* | [*ACR*](http://www.genecards.org/cgi-bin/carddisp.pl?gene=ACR) | Acrosin | 9 | 8h down |
| *NM_054025* | [*B3GAT1*](http://www.genecards.org/cgi-bin/carddisp.pl?gene=B3GAT1) | Beta-1,3-glucuronyltransferase 1 (glucuronosyltransferase P) | 9 | 8h down |
| *NM_001713* | [*BHMT*](http://www.genecards.org/cgi-bin/carddisp.pl?gene=BHMT) | Betaine-homocysteine methyltransferase | 9 | 8h down |
| *AB040958* | [*BTBD7*](http://www.genecards.org/cgi-bin/carddisp.pl?gene=BTBD7) | BTB (POZ) domain containing 7 | 9 | 8h down |
| *BC015423* | [*FAM14B*](http://www.genecards.org/cgi-bin/carddisp.pl?gene=FAM14B) | Family with sequence similarity 14, member B | 9 | 8h down |
| *U66589* | [*FAM69A*](http://www.genecards.org/cgi-bin/carddisp.pl?gene=FAM69A)  [*RPL5*](http://www.genecards.org/cgi-bin/carddisp.pl?gene=RPL5) | Ribosomal protein L5 | 9 | 8h down |
| *NM_000162* | [*GCK*](http://www.genecards.org/cgi-bin/carddisp.pl?gene=GCK) | Glucokinase (hexokinase 4, maturity onset diabetes of the young 2) | 9 | 8h down |
| *NM_001549* | [*IFIT3*](http://www.genecards.org/cgi-bin/carddisp.pl?gene=IFIT3) | Interferon-induced protein with tetratricopeptide repeats 3 | 9 | 8h down |
| *NM_012311* | [*KIN*](http://www.genecards.org/cgi-bin/carddisp.pl?gene=KIN) | KIN, antigenic determinant of recA protein homolog (mouse) | 9 | 8h down |
| *U96173* | [*ONECUT1*](http://www.genecards.org/cgi-bin/carddisp.pl?gene=ONECUT1) | One cut domain, family member 1 | 9 | 8h down |
| *NM_002841* | [*PTPRG*](http://www.genecards.org/cgi-bin/carddisp.pl?gene=PTPRG) | Protein tyrosine phosphatase, receptor type, G | 9 | 8h down |
| *NM_002967* | [*SAFB*](http://www.genecards.org/cgi-bin/carddisp.pl?gene=SAFB) | Scaffold attachment factor B | 9 | 8h down |
| *AK056015* | [*UBE3B*](http://www.genecards.org/cgi-bin/carddisp.pl?gene=UBE3B) | Ubiquitin protein ligase E3B | 9 | 8h down |
| *AF190748* | [*ENSG00000183673*](http://www.genecards.org/cgi-bin/carddisp.pl?gene=ENSG00000183673&search=AF190748) | FGF2-associated protein GAFA1 (GAFA1) | 9 | 8h down |
| *AK021717* | *N/A* |  | 9 | 8h down |
| *AL021327* | *N/A* |  | 9 | 8h down |
| *D87009* | *N/A* |  | 9 | 8h down |
| *NM_032950* | [*MMP28*](http://www.genecards.org/cgi-bin/carddisp.pl?gene=MMP28&search=NM_032950) | matrix metallopeptidase 28 | 9 | 8h down |
| *AB033061* | [*ARID1B*](http://www.genecards.org/cgi-bin/carddisp.pl?gene=ARID1B) | AT rich interactive domain 1B (SWI1-like) | 10 | 10h down |
| *NM_032127* | [*C11orf56*](http://www.genecards.org/cgi-bin/carddisp.pl?gene=C11orf56) | Chromosome 11 open reading frame 56 | 10 | 10h down |
| *AK023008* | [*C14orf135*](http://www.genecards.org/cgi-bin/carddisp.pl?gene=C14orf135) | Chromosome 14 open reading frame 135 | 10 | 10h down |
| *NM_015434* | [*C1orf73*](http://www.genecards.org/cgi-bin/carddisp.pl?gene=C1orf73) | Chromosome 1 open reading frame 73 | 10 | 10h down |
| *NM_016940* | [*C21orf6*](http://www.genecards.org/cgi-bin/carddisp.pl?gene=C21orf6) | Chromosome 21 open reading frame 6 | 10 | 10h down |
| *NM_001844* | [*COL2A1*](http://www.genecards.org/cgi-bin/carddisp.pl?gene=COL2A1) | Collagen, type II, alpha 1 (primary osteoarthritis, spondyloepiphyseal dysplasia, congenital) | 10 | 10h down |
| *NM_020248* | [*CTNNBIP1*](http://www.genecards.org/cgi-bin/carddisp.pl?gene=CTNNBIP1) | Catenin, beta interacting protein 1 | 10 | 10h down |
| *NM_032036* | [*FAM14A*](http://www.genecards.org/cgi-bin/carddisp.pl?gene=FAM14A) | Family with sequence similarity 14, member A | 10 | 10h down |
| *AK056199* | [*FUT10*](http://www.genecards.org/cgi-bin/carddisp.pl?gene=FUT10) | Fucosyltransferase 10 (alpha (1,3) fucosyltransferase) | 10 | 10h down  Top of Form |
| *BC014851* | [*LFNG*](http://www.genecards.org/cgi-bin/carddisp.pl?gene=LFNG) | Lunatic fringe homolog (Drosophila) | 10 | 10h down |
| *NM_015929* | [*LIPT1*](http://www.genecards.org/cgi-bin/carddisp.pl?gene=LIPT1) | Lipoyltransferase 1 | 10 | 10h down |
| *AK026720* | [*LOC283537*](http://www.genecards.org/cgi-bin/carddisp.pl?gene=LOC283537) | Hypothetical protein LOC283537 | 10 | 10h down |
| *AK056736* | [*MBTPS2*](http://www.genecards.org/cgi-bin/carddisp.pl?gene=MBTPS2) | Membrane-bound transcription factor peptidase, site 2 | 10 | 10h down  Bottom of Form |
| *NM_004530* | [*MMP2*](http://www.genecards.org/cgi-bin/carddisp.pl?gene=MMP2) | Matrix metallopeptidase 2 (gelatinase A, 72kDa gelatinase, 72kDa type IV collagenase) | 10 | 10h down |
| *NM_053050* | [*MRPL53*](http://www.genecards.org/cgi-bin/carddisp.pl?gene=MRPL53) | Mitochondrial ribosomal protein L53 | 10 | 10h down |
| *NM_013330* | [*NME7*](http://www.genecards.org/cgi-bin/carddisp.pl?gene=NME7) | Non-metastatic cells 7, protein expressed in (nucleoside-diphosphate kinase) | 10 | 10h down |
| *NM_024165* | [*PHF1*](http://www.genecards.org/cgi-bin/carddisp.pl?gene=PHF1) | PHD finger protein 1 | 10 | 10h down |
| *NM_006221* | [*PIN1*](http://www.genecards.org/cgi-bin/carddisp.pl?gene=PIN1) | Protein (peptidylprolyl cis/trans isomerase) NIMA-interacting 1 | 10 | 10h down |
| *NM_000542* | [*SFTPB*](http://www.genecards.org/cgi-bin/carddisp.pl?gene=SFTPB) | Surfactant, pulmonary-associated protein B | 10 | 10h down |
| *NM_003745* | [*SOCS1*](http://www.genecards.org/cgi-bin/carddisp.pl?gene=SOCS1) | Suppressor of cytokine signaling 1 | 10 | 10h down |
| *NM_007269* | [*STXBP3*](http://www.genecards.org/cgi-bin/carddisp.pl?gene=STXBP3) | Syntaxin binding protein 3 | 10 | 10h down |
| *AB040918* | [*ZNF406*](http://www.genecards.org/cgi-bin/carddisp.pl?gene=ZNF406) | Zinc finger protein 406 | 10 | 10h down |
| *NM_018337* | [*ZNF444*](http://www.genecards.org/cgi-bin/carddisp.pl?gene=ZNF444) | Zinc finger protein 444 | 10 | 10h down |
| *AK000879* | [*ENSG00000176593*](http://www.genecards.org/cgi-bin/carddisp.pl?gene=ENSG00000176593&search=AK000879) | CDNA FLJ37429 fis, clone BRAWH2001666 | 10 | 10h down |
| *AK023632* | [*VPS54*](http://www.genecards.org/cgi-bin/carddisp.pl?gene=VPS54&search=AK023632) | vacuolar protein sorting 54 (yeast) | 10 | 10h down |
| *BC010051* | *N/A* |  | 10 | 10h down |
| *BC015903* | *N/A* | Full length insert cDNA clone YA80A03 | 10 | 10h down |
