# Supplementary material for: Systems biology approach for mapping the response of human urothelial cells to infection by Enterococcus faecalis
Source: BMC Bioinformatics. 2007 Nov 1;8(Suppl 7):S2. doi: 10.1186/1471-2105-8-S7-S2 (PMC2099488; doi:10.1186/1471-2105-8-S7-S2)
Supplement: Additional file 17 — Full list of 192 HV genes in cluster 1. GenBank accession numbers, gene names and description provided. [file 1471-2105-8-S7-S2-S17.doc]

**Table 2 suppl. Full list of 192 HV genes in cluster 1.**

| **Gene Name** | **Entrez Gene** | **GenBank Accession** | **Description** |
| --- | --- | --- | --- |
| ACTR2 | 10097 | AK025051 | ARP2 actin-related protein 2 homolog (yeast) |
| ADCY7 | 113 | NM_001114 | Adenylate cyclase 7 |
| AGTRAP | 57085 | NM_020350 | Angiotensin II receptor-associated protein |
| APPL | 26060 | NM_012096 | Adaptor protein containing pH domain, PTB domain and leucine zipper motif 1 |
| ARHGEF19 | 128272 | AL137736 | Rho guanine nucleotide exchange factor (GEF) 19 |
| ARL3 | 403 | NM_004311 | ADP-ribosylation factor-like 3 |
| ASCC2 | 84164 | NM_032204 | Activating signal cointegrator 1 complex subunit 2 |
| ASNS | 440 | NM_001673 | Asparagine synthetase |
| ATP6V1F | 9296 | NM_004231 | ATPase, H+ transporting, lysosomal 14kDa, V1 subunit F |
| BCL7B | 9275 | NM_020179 | B-cell CLL/lymphoma 7B |
| BOC | 91653 | AK056359 | Boc homolog (mouse) |
| BRWD1 | 54014 | AJ238555 | Bromodomain and WD repeat domain containing 1 |
| BXDC5 | 80135 | NM_025065 | Brix domain containing 5 |
| C12orf23 | 90488 | AK001731 | Chromosome 12 open reading frame 23 |
| C14orf80 | 283643 | AK024506 | Chromosome 14 open reading frame 80 |
| C16orf59 | 80178 | BC008882 | Chromosome 16 open reading frame 59 |
| C16orf72 | 29035 | AK057657 | Chromosome 16 open reading frame 72 |
| C1orf27 | 54953 | NM_022375 | Chromosome 1 open reading frame 27 |
| C20orf121 | 79183 | AK056178 | Chromosome 20 open reading frame 121 |
| C6orf111 | 25957 | AK027751 | Chromosome 6 open reading frame 111 |
| C6orf120 | 387263 | AF055030 | Chromosome 6 open reading frame 120 |
| C7orf23 | 79161 | NM_024315 | Chromosome 7 open reading frame 23 |
| C7orf25 | 79020 | NM_024054 | Chromosome 7 open reading frame 25 |
| CALD1 | 800 | AJ223812 | Caldesmon 1 |
| CBARA1 | 10367 | NM_006077 | Calcium binding atopy-related autoantigen 1 |
| CCDC50 | 152137 | AK055652 | Coiled-coil domain containing 50 |
| CD320 | 51293 | NM_016579 | CD320 molecule |
| CDK2AP2 | 10263 | NM_005851 | CDK2-associated protein 2 |
| CDYL | 9425 | NM_004824 | Chromodomain protein, Y-like |
| CETN3 | 1070 | NM_004365 | Centrin, EF-hand protein, 3 (CDC31 homolog, yeast) |
| CGI-38 | 51673 | NM_016140 | Brain specific protein |
| CNP | 1267 | NM_033133 | 2',3'-cyclic nucleotide 3' phosphodiesterase |
| COQ2 | 27235 | BC008804 | Coenzyme Q2 homolog, prenyltransferase (yeast) |
| CORIN | 10699 | NM_006587 | Corin, serine peptidase |
| CPM | 1368 | AF368463 | Carboxypeptidase M |
| CRSP3 | 9439 | NM_004830 | Cofactor required for Sp1 transcriptional activation, subunit 3, 130kDa |
| CUEDC2 | 79004 | NM_024040 | CUE domain containing 2 |
| DGAT2 | 84649 | NM_032564 | Diacylglycerol O-acyltransferase homolog 2 (mouse) |
| DGKE | 8526 | NM_003647 | Diacylglycerol kinase, epsilon 64kDa |
| DNAJA2 | 10294 | NM_005880 | DnaJ (Hsp40) homolog, subfamily A, member 2 |
| EDG4 | 9170 | NM_004720 | Endothelial differentiation, lysophosphatidic acid G-protein-coupled receptor, 4 |
| EGR1 | 1958 | NM_001964 | Early growth response 1 |
| EHMT2 | 10919 | NM_006709 | Euchromatic histone-lysine N-methyltransferase 2 |
| ERAL1 | 26284 | AY007435 | Era G-protein-like 1 (E. coli) |
| FAM125A | 93343 | AK057057 | Family with sequence similarity 125, member A |
| FAM38B | 63895 | AK056572 | Family with sequence similarity 38, member B |
| FAM39B | 375260 | BC015400 | Family with sequence similarity 39, member B |
| FANCL | 55120 | NM_018062 | Fanconi anemia, complementation group L |
| FBXL11 | 22992 | NM_012308 | F-box and leucine-rich repeat protein 11 |
| FBXO10 | 26267 | BC012155 | F-box protein 10 |
| FLT1 | 2321 | AF339822 | Fms-related tyrosine kinase 1 (vascular endothelial growth factor/vascular permeability factor receptor) |
| GALNT10 | 55568 | AK023815 | UDP-N-acetyl-alpha-D-galactosamine:polypeptide N-acetylgalactosaminyltransferase 10 (GalNAc-T10) |
| GALR3 | 8484 | NM_003614 | Galanin receptor 3 |
| GBA | 2629 | D13287 | Glucosidase, beta; acid (includes glucosylceramidase) |
| GDPD5 | 81544 | NM_030792 | Glycerophosphodiester phosphodiesterase domain containing 5 |
| GTF2H3 | 2967 | NM_001516 | General transcription factor IIH, polypeptide 3, 34kDa |
| H2AFY2 | 55506 | NM_018649 | H2A histone family, member Y2 |
| HAX1 | 10456 | NM_006118 | HCLS1 associated protein X-1 |
| HDAC11 | 79885 | NM_024827 | Histone deacetylase 11 |
| HIRIP3 | 8479 | NM_003609 | HIRA interacting protein 3 |
| HIST1H2BM | 8342 | NM_003521 | Histone cluster 1, H2bm |
| HOOK2 | 29911 | NM_013312 | Hook homolog 2 (Drosophila) |
| ICK | 22858 | NM_014920 | Intestinal cell (MAK-like) kinase |
| IFT172 | 26160 | AL110218 | Intraflagellar transport 172 homolog (Chlamydomonas) |
| IKIP | 121457 | AK055613 | IKK interacting protein |
| IKZF1 | 10320 | NM_006060 | IKAROS family zinc finger 1 (Ikaros) |
| IL1F9 | 56300 | NM_019618 | Interleukin 1 family, member 9 |
| ITFG1 | 81533 | BC015844 | Integrin alpha FG-GAP repeat containing 1 |
| ITGB7 | 3695 | NM_000889 | Integrin, beta 7 |
| JUNB | 3726 | NM_002229 | Jun B proto-oncogene |
| KATNAL2 | 83473 | AL512748 | Katanin p60 subunit A-like 2 |
| KCNMB4 | 27345 | AL137510 | Potassium large conductance calcium-activated channel, subfamily M, beta member 4 |
| KIAA0146 | 23514 | D63480 | KIAA0146 |
| KIAA1219 | 57148 | AK026771 | KIAA1219 |
| KLHDC4 | 54758 | AK057211 | Kelch domain containing 4 |
| KLHL10 | 317719 | AK057224 | Kelch-like 10 (Drosophila) |
| KLHL23 | 151230 | BC016950 | Kelch-like 23 (Drosophila) |
| KNS2 | 3831 | AL133587 | Kinesin 2 |
| LAMA4 | 3910 | NM_002290 | Laminin, alpha 4 |
| LCK | 3932 | NM_005356 | Lymphocyte-specific protein tyrosine kinase |
| LOC115110 | 115110 | AL359943 | Hypothetical protein LOC115110 |
| LOC197350 | 197350 | AF098666 | Hypothetical protein LOC197350 |
| LOC375010 | 375010 | BC012753 | Hypothetical LOC375010 |
| LOC440330 | 440330 | BC004968 | Hypothetical LOC440330 |
| LOC642776 | 642776 | BC003645 | Hypothetical protein LOC642776 |
| LRRC51 | 220074 | BC012855 | Leucine rich repeat containing 51 |
| MACF1 | 23499 | AF317696 | Microtubule-actin crosslinking factor 1 |
| MARS2 | 92935 | BC009115 | Methionine-tRNA synthetase 2 (mitochondrial) |
| MDFI | 4188 | NM_005586 | MyoD family inhibitor |
| ME2 | 4200 | NM_002396 | Malic enzyme 2, NAD(+)-dependent, mitochondrial |
| MGC14327 | 94107 | NM_053045 | Hypothetical protein MGC14327 |
| MGC3260 | 78993 | BC000073 | Hypothetical protein MGC3260 |
| MREG | 55686 | NM_018000 | Melanoregulin |
| MRPS6 | 64968 | AK055913 | Mitochondrial ribosomal protein S6 |
| MS4A3 | 932 | L35848 | Membrane-spanning 4-domains, subfamily A, member 3 (hematopoietic cell-specific) |
| MYO1B | 4430 | AK000160 | Myosin IB |
| NBR1 | 4077 | NM_031858 | Neighbor of BRCA1 gene 1 |
| NCAPH | 23397 | D38553 | Non-SMC condensin I complex, subunit H |
| NDUFA7 | 4701 | NM_005001 | NADH dehydrogenase (ubiquinone) 1 alpha subcomplex, 7, 14.5kDa |
| NR0B1 | 190 | NM_000475 | Nuclear receptor subfamily 0, group B, member 1 |
| NUP188 | 23511 | BC005407 | Nucleoporin 188kDa |
| OCIAD1 | 54940 | AK024302 | OCIA domain containing 1 |
| OPN1SW | 611 | NM_001708 | Opsin 1 (cone pigments), short-wave-sensitive (color blindness, tritan) |
| PDCD2L | 84306 | NM_032346 | Programmed cell death 2-like |
| PEX3 | 8504 | NM_003630 | Peroxisomal biogenesis factor 3 |
| PIP5K1A | 8394 | NM_003557 | Phosphatidylinositol-4-phosphate 5-kinase, type I, alpha |
| PLA2G12B | 84647 | NM_032562 | Phospholipase A2, group XIIB |
| PLIN | 5346 | NM_002666 | Perilipin |
| PMS2L5 | 5383 | D38501 | Postmeiotic segregation increased 2-like 5 |
| PNKP | 11284 | NM_007254 | Polynucleotide kinase 3'-phosphatase |
| PNPT1 | 87178 | BC000862 | Polyribonucleotide nucleotidyltransferase 1 |
| POLR2K | 5440 | NM_005034 | Polymerase (RNA) II (DNA directed) polypeptide K, 7.0kDa |
| POMGNT1 | 55624 | NM_017739 | Protein O-linked mannose beta1,2-N-acetylglucosaminyltransferase |
| PRDM1 | 639 | NM_001198 | PR domain containing 1, with ZNF domain |
| PSD3 | 23362 | NM_015310 | Pleckstrin and Sec7 domain containing 3 |
| PSMB5 | 5693 | NM_002797 | Proteasome (prosome, macropain) subunit, beta type, 5 |
| PTCRA | 171558 | U36759 | Pre T-cell antigen receptor alpha |
| PTGER1 | 5731 | NM_000955 | Prostaglandin E receptor 1 (subtype EP1), 42kDa |
| PTPRN2 | 5799 | AK057320 | Protein tyrosine phosphatase, receptor type, N polypeptide 2 |
| PTPRZ1 | 5803 | NM_002851 | Protein tyrosine phosphatase, receptor-type, Z polypeptide 1 |
| QTRTD1 | 79691 | NM_024638 | Queuine tRNA-ribosyltransferase domain containing 1 |
| RAB6IP1 | 23258 | AL117448 | RAB6 interacting protein 1 |
| RGR | 5995 | BC008094 | Retinal G protein coupled receptor |
| ROCK2 | 9475 | NM_004850 | Rho-associated, coiled-coil containing protein kinase 2 |
| RPL35A | 6165 | AK021571 | Ribosomal protein L35a |
| RPS6KB1 | 6198 | AK026730 | Ribosomal protein S6 kinase, 70kDa, polypeptide 1 |
| SART1 | 9092 | NM_005146 | Squamous cell carcinoma antigen recognised by T cells |
| SCAND1 | 51282 | NM_016558 | SCAN domain containing 1 |
| SCRN2 | 90507 | BC002980 | Secernin 2 |
| SF3A1 | 10291 | NM_005877 | Splicing factor 3a, subunit 1, 120kDa |
| SFRS5 | 6430 | NM_006925 | Splicing factor, arginine/serine-rich 5 |
| SLITRK6 | 84189 | NM_032229 | SLIT and NTRK-like family, member 6 |
| SMARCD1 | 6602 | NM_003076 | SWI/SNF related, matrix associated, actin dependent regulator of chromatin, subfamily d, member 1 |
| SNIP1 | 79753 | NM_024700 | Smad nuclear interacting protein 1 |
| SOCS4 | 122809 | AK056685 | Suppressor of cytokine signaling 4 |
| STRN4 | 29888 | NM_013403 | Striatin, calmodulin binding protein 4 |
| SULT1E1 | 6783 | NM_005420 | Sulfotransferase family 1E, estrogen-preferring, member 1 |
| TAF12 | 6883 | NM_005644 | TAF12 RNA polymerase II, TATA box binding protein (TBP)-associated factor, 20kDa |
| TCF3 | 6929 | M31523 | Transcription factor 3 (E2A immunoglobulin enhancer binding factors E12/E47) |
| TCP10L | 140290 | AK058078 | T-complex 10 (mouse)-like |
| TEKT1 | 83659 | NM_053285 | Tektin 1 |
| THAP7 | 80764 | NM_030573 | THAP domain containing 7 |
| TIE1 | 7075 | NM_005424 | Tyrosine kinase with immunoglobulin-like and EGF-like domains 1 |
| TM9SF3 | 56889 | NM_020123 | Transmembrane 9 superfamily member 3 |
| TMEM15 | 22845 | NM_014908 | Transmembrane protein 15 |
| TMTC4 | 84899 | NM_032813 | Transmembrane and tetratricopeptide repeat containing 4 |
| TP73 | 7161 | NM_005427 | Tumor protein p73 |
| TRIM5 | 85363 | NM_033034 | Tripartite motif-containing 5 |
| TRIM56 | 81844 | BC005847 | Tripartite motif-containing 56 |
| TRMU | 55687 | NM_018006 | TRNA 5-methylaminomethyl-2-thiouridylate methyltransferase |
| UBE2J2 | 118424 | NM_058167 | Ubiquitin-conjugating enzyme E2, J2 (UBC6 homolog, yeast) |
| ULK1 | 8408 | NM_003565 | Unc-51-like kinase 1 (C. elegans) |
| UNC45A | 55898 | BC006214 | Unc-45 homolog A (C. elegans) |
| USP31 | 57478 | AK057491 | Ubiquitin specific peptidase 31 |
| ZDHHC4 | 55146 | NM_018106 | Zinc finger, DHHC-type containing 4 |
| ZFYVE1 | 53349 | AB046809 | Zinc finger, FYVE domain containing 1 |
| ZNF641 | 121274 | AK055857 | Zinc finger protein 641 |
| ZNF649 | 65251 | NM_023074 | Zinc finger protein 649 |
|  |  | NM_032585 | |
|  |  | AF017336 | |
|  |  | NM_024984 | |
|  |  | BC006438 | CDNA clone MGC:13162 IMAGE:3010103 |
|  |  | NM_032903 | |
|  |  | D00267 |  |
|  |  | AK024911 | |
|  |  | AC003989 | |
|  |  | BC007937 | |
|  |  | AK055347 | CDNA FLJ30785 fis, clone FEBRA2000901 |
|  |  | AK022389 | |
|  |  | BC010906 | |
|  |  | NM_014131 | |
|  |  | AK021643 | |
|  |  | AK057861 | |
|  |  | J05582 |  |
|  |  | BC001618 | |
|  |  | NM_031423 | |
|  |  | AL024509 | |
|  |  | NM_033424 | |
|  |  | NM_032931 | |
|  |  | NM_024977 | |
|  |  | NM_014136 | |
|  |  | NM_019081 | |
|  |  | AK057515 | CDNA FLJ32953 fis, clone TESTI2008099 |
|  |  | AK056826 | CDNA FLJ32264 fis, clone PROST1000383 |
|  |  | AK055812 | |
|  |  | NM_024998 | |
|  |  | NM_016430 | |
|  |  | AK054960 | |
|  |  | NM_032297 | |
|  |  | AK021952 | CDNA FLJ11890 fis, clone HEMBA1007256 |
|  |  | NM_005083 | |
|  |  | X72309 |  |
