# Supplementary material for: Systems biology approach for mapping the response of human urothelial cells to infection by Enterococcus faecalis
Source: BMC Bioinformatics. 2007 Nov 1;8(Suppl 7):S2. doi: 10.1186/1471-2105-8-S7-S2 (PMC2099488; doi:10.1186/1471-2105-8-S7-S2)
Supplement: Additional file 18 — Genes from expanded cluster 1 assembled in networks and their top functions/canonical pathways. Gene names in bold are "focus genes" identified from clusters formed from VHV genes. Non-expressed genes were manually removed from the networks. Statistically significant top functions and canonical pathways are identified by IPA. [file 1471-2105-8-S7-S2-S18.doc]

**Table 3 suppl. Genes from expanded cluster 1 assembled in networks and their top functions/canonical pathways.**

| # | **Gene names** | **Network’s top functions** | **Canonical pathways** |
| --- | --- | --- | --- |
| 1 | **ARL3**, **ATP6V1F,** BCL2L1, CALB1, CASP8AP2, CDK2, CDKN2A, DDR1, **EGR1**, **FANCL**, **FLT1**, FMR1, **GALNT10**, **GOLGA4**, HPSE, IL1B, **JUNB**, KAP, MAP3K7IP3, **MS4A3**, **PNPT1**, PTGES, SLC25A4, SRPK1, ST8SIA1, **SULT1E1**, **TIE1**, TRAF2, UBE2A (includes EG:7319), **UBE2J2**, **ZNFN1A1** | Cell Cycle, Cell Death, Connective Tissue Disorders, Cell Morphology, Cancer | PPAR Signaling, p38 MAPK Signaling, NF-κB Signaling, IL-6 Signaling, B Cell Receptor Signaling |
| 2 | **ACTR2**, ARPC2, **ASNS**, **BRRN1**, **CALD1**, **CCL7**, **CGI-38**, COL6A2, **CORIN**, **CPM**, **GRB10**, HAS1, IARS (includes EG:3376), IL8, IL13, **ITGB7**, **NOXA1**, PF4, **PTGER1**, **PTPRZ1**, SCAP2, SRC, TGFB1, TNS1 | Cellular Movement, Cell Death, Immune and Lymphatic System Development and Function, Immune Response | Integrin Signaling, Ephrin Receptor Signaling,Wnt/β-catenin Signaling, Chemokine Signaling, Actin Cytosceleton Signaling |
| 3 | ARF4, **CNP**, DNTT, EGFR, **HAX1**, **HNRPM**, JUN, **LAMA4**, LAMP2, **MACF1**, MYC, **MYO1B**, PCBP2, PFKFB1, **PIP5K1A**, PKD2 (includes EG:5311), **PRDM1**, PRKACA, **PTCRA**, **PTPRN2**, **ROCK2**, RPS7, RPS23, **SART1**, SUMO2 (includes EG:6613), **TCF3**, ZAK | Cell Cycle, Cancer, Tumor Morphology, Cellular Development, Cellular Growth and Proliferation | Actin Cytosceleton Signaling, Wnt/β-catenin Signaling |
| 4 | AGT, **AGTRAP**, AKT1, **APPL**, **CRSP3**, EDG1, ESR1, HIPK3, MPG, NCOA7, NCOA4 (includes EG:8031), NCOR1, **NR0B1**, NR3C1, **PLIN**, PPARG, PRMT2, RELA, **RPS6KB1**, **SCAND1**, SDPR, **SF3A1**, **SMARCD1**, **SNIP1 (includes EG:79753)**, **THAP7**, TRIB3, **UNC45A** | Gene Expression, Cell Signaling, Small Molecule Biochemistry | Estrogen Receptor Signaling, PTEN Signaling, PPAR Signaling, IL-4 Signaling |
| 5 | **ADCY7**, CD160, **CDK2AP2**, **EDG4**, ERCC3, FCGR1B, **GTF2H3**, IFNG, **IL1F9**, LARGE, **MUC1**, **NBR1**, **PNKP**, PPP1R13B, SDCBP, **SFRS5**, TP53, **TP73**, ULK1 | Cancer, Cell Death, Sceletal and Muscular Disorders | Estrogen Receptor Signaling |
| 6 | CBX4, **CDYL**, CETN3, CTBP1, EHMT1, **EHMT2**, **ICK**, IL15, JRK, KITLG (includes EG:4254), **LCK**, LYPLA1, **MDFI (includes EG:4188)**, **POLR2K**, PRG1 (includes EG:5552), **PSMB5**, RPL7, RPS7, SNAI2, **TAF12**, **TRIM5** | Cellular Development, Hematological System Development and Function, Immune and Lymphatic System Development and Function, Immune Response | Estrogen Receptor Signaling |
